# Heat shock induces premature transcript termination and reconfigures the human transcriptome

**DOI:** 10.1016/j.molcel.2022.01.007

**Published:** 2022-04-21

**Authors:** Simona Cugusi, Richard Mitter, Gavin P. Kelly, Jane Walker, Zhong Han, Paola Pisano, Michael Wierer, Aengus Stewart, Jesper Q. Svejstrup

**Affiliations:** 1 Mechanisms of Transcription Laboratory, The Francis Crick Institute, 1 Midland Road, London NW1 1AT, UK; 2 Bioinformatics and Biostatistics, The Francis Crick Institute, 1 Midland Road, London NW1 1AT, UK; 3 Department of Cellular and Molecular Medicine, Panum Institute, Blegdamsvej 3B, University of Copenhagen, 2200 Copenhagen N, Denmark; 4 Proteomics Research Infrastructure, Panum Institute, Blegdamsvej 3B, University of Copenhagen, 2200 Copenhagen N, Denmark

**Keywords:** heat shock, transcriptional repression, U1 snRNA, telescripting, alternative polyadenylation, cryptic polyadenylation sites, premature termination, SCAF4, SCAF8, CPSF3, CDK9, pTEFb, TT-seq, elongation, pause release

## Abstract

The heat shock (HS) response involves rapid induction of HS genes, whereas transcriptional repression is established more slowly at most other genes. Previous data suggested that such repression results from inhibition of RNA polymerase II (RNAPII) pause release, but here, we show that HS strongly affects other phases of the transcription cycle. Intriguingly, while elongation rates increase upon HS, processivity markedly decreases, so that RNAPII frequently fails to reach the end of genes. Indeed, HS results in widespread premature transcript termination at cryptic, intronic polyadenylation (IPA) sites near gene 5′-ends, likely via inhibition of U1 telescripting. This results in dramatic reconfiguration of the human transcriptome with production of new, previously unannotated, short mRNAs that accumulate in the nucleus. Together, these results shed new light on the basic transcription mechanisms induced by growth at elevated temperature and show that a genome-wide shift toward usage of IPA sites can occur under physiological conditions.

## Highlights

• Transcription downregulation during HS is caused by premature termination
• Transcription elongation rate increases markedly during HS
• HS disrupts telescripting and activates cryptic poly(A) sites in introns
• HS results in widespread synthesis of new, short mRNAs species

## Graphical abstract

**Figure:**
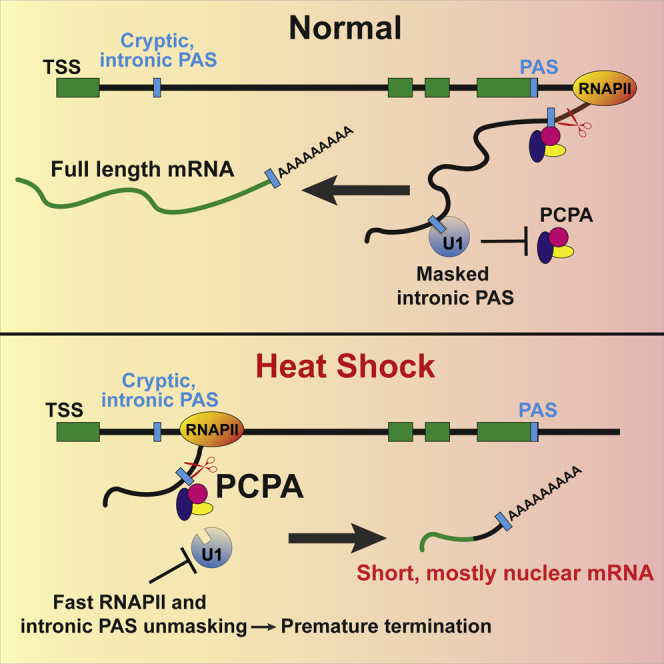

## Teaser

Cugusi et al. show that the elongation rate is dramatically increased during heat shock, while the global downregulation of transcription observed under these conditions is caused by premature termination at cryptic PAS in introns, which results in the synthesis of a large number of new, short mRNA species.

## Introduction

The ability to respond rapidly to stress is crucial for cellular, tissue, and organismal survival. The heat shock response (HSR) is a highly conserved cellular program induced by the exposure to a variety of environmental stressors, among which heat shock (HS) was the first observed and the most studied (Lindquist, 1986; Morimoto, 1998). Following HS, a subset of genes, known as the HS genes, are rapidly induced by heat shock factor (HSF) to help maintain protein homeostasis and ensure cell survival (Vihervaara et al., 2018), not least through the production of protein chaperones to negate heat-induced protein unfolding (Lindquist and Craig, 1988; Schopf et al., 2017). However, several lines of evidence suggest that the HSR has a broad range of physiological roles (Akerfelt et al., 2010), and a growing body of research indicates that it is co-opted by cancer cells to support malignancy and that it also plays a role in the pathogenesis of neurodegenerative diseases (Gomez-Pastor et al., 2018).

The mechanism underlying HS-induced gene activation has been extensively studied, and the HSR has generally proven to be a helpful model to more generally understand the dynamics of transcription (Wu et al., 2003; Ardehali et al., 2009; Guertin et al., 2010; Chen et al., 2017; Liang et al., 2018; Vihervaara et al., 2018; Gressel et al., 2019). Interestingly, while the upregulation of HS genes has been recognized and studied for decades, a fascinating and still poorly understood aspect of the HSR is the parallel transcriptional repression of a large portion of the genome (Jamrich et al., 1977; Gasch et al., 2000; Mahat et al., 2016). HS-associated, global gene repression has been documented in different species, and in recent years efforts have been made to reveal the underlying mechanism (Allen et al., 2004; Mariner et al., 2008; Mahat et al., 2016; Vihervaara et al., 2017; Aprile-Garcia et al., 2019; Rawat et al., 2021). Specific short interspersed nuclear elements (SINEs), which are upregulated during HS, are capable of binding directly to RNA polymerase II (RNAPII) and preventing the formation of the pre-initiation complex (Allen et al., 2004; Kettenberger et al., 2006; Mariner et al., 2008). This led to the suggestion that repression by HS might occur at initiation. However, this mechanism does not appear to be a widespread mode of action. In fact, subsequent genome-wide studies indicate that at most HS-repressed genes, transcription starts normally but progression through the gene body is halted by an unknown mechanism (Hieda et al., 2005; Teves and Henikoff, 2011; Mahat et al., 2016; Vihervaara et al., 2017). The prevalent current models resulting from this work suggest that HS somehow prevents RNAPII from transitioning into productive elongation, resulting in increased promoter-proximal pausing. Interestingly, however, the approaches typically used so far have arguably not been ideal for analyzing the entire transcription cycle, and the results obtained have yet to present a compelling molecular model for HS-induced repression of gene expression.

While regulation of transcription in general was initially thought to occur almost exclusively at initiation, research over the last two decades has shown that regulation of other phases of the transcription cycle can affect gene expression as well (Bentley, 2014; Kamieniarz-Gdula and Proudfoot, 2019). For example, most RNAPII genes are subject to a low level of premature 3′-end cleavage and polyadenylation (PCPA) at cryptic, intronic polyadenylation (IPA) sites. It was first thought that only polyadenylation sites (PASs) in terminal exons or 3′ untranslated regions (UTRs) are utilized, but more recent work shows PCPA occurring across genes and particularly at IPA sites, depending on cell type, growth conditions, and sometimes disease state (Wang et al., 2008, 2018; Gruber and Zavolan, 2019). Intriguingly, interference with U1 snRNA (U1) base pairing to 5′-splice sites, necessary for U1 function in splicing, was found to cause widespread PCPA and frequent premature transcription termination (Kaida et al., 2010; Berg et al., 2012). Indeed, U1’s suppression of PCPA activity and transcription termination is crucial for allowing the production of long (and full-length) transcripts from thousands of vertebrate genes, a process termed telescripting (Venters et al., 2019). Whether inhibition of telescripting also controls the level of gene expression under physiological conditions has remained unknown.

To better understand the mechanisms employed by cells to repress gene expression after HS, we investigated whether other phases of the transcription cycle such as elongation and termination are affected as well. Here, we present evidence that transcription downregulation during HS is mainly achieved by premature transcript termination through disruption of telescripting. This results in dramatic reconfiguration of the human transcriptome with thousands of new, short mRNAs being produced.

## Results

### HS results in defective transcription elongation

To obtain an expansive view of the transcription changes occurring during HS in human cells, we analyzed nascent RNA synthesis using transient transcriptome sequencing (TT_chem_-seq) (Schwalb et al., 2016; Gregersen et al., 2020). We initially used colony survival assays to identify the longest time of continuous HS that minimally affects cell viability (Figure S1). These experiments were carried out in parental and HSF1 knockdown MRC5-VA cell lines, using mild HS conditions for up to 4 h. The results led us to perform the TT_chem_-seq experiment after exposing cells to 43°C for 2 h.

**Figure 1:**
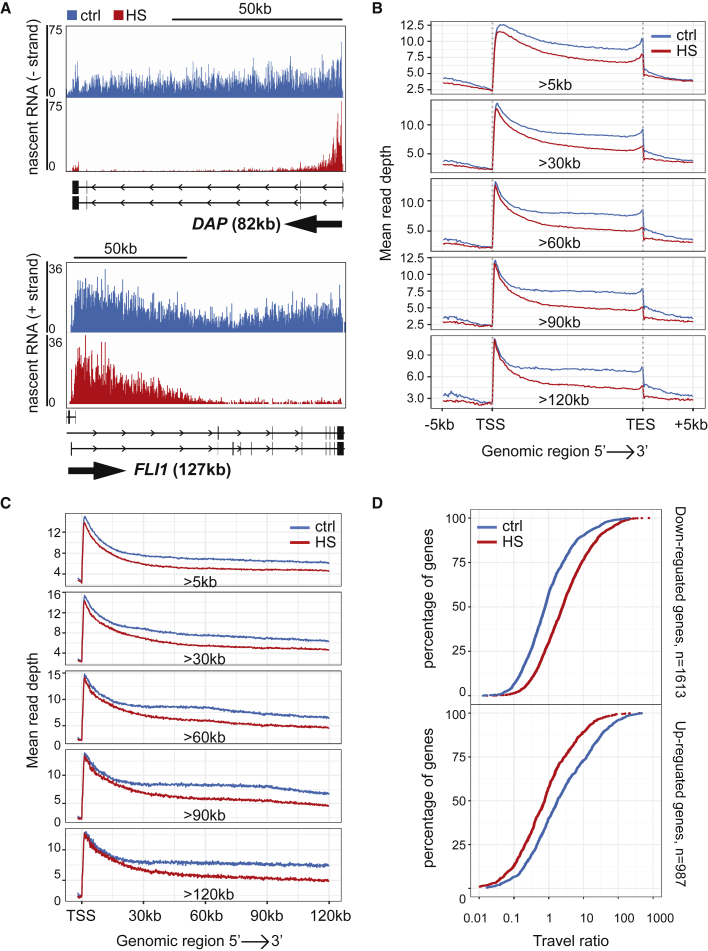
Heat shock results in defective transcript elongation (A) IGV genome browser views of TT_chem_-seq for the *DAP* and *FLI1* genes. Arrows indicate the direction of transcription. (B) Metagene profiles of TT_chem_-seq stratified by gene length. (C) Coverage plots of TT_chem_-seq stratified by gene length. TES, 3′ transcript end site (poly(A) site). (D) Travel ratios of genes downregulated by HS (upper panel) and genes upregulated by HS (lower panel). See also Figures S1–S3. Genome-wide visualizations in the figures of this report generally represent at least two merged biological replicates.

Single gene profiles identified by manual inspection of the data using genome browser nicely illustrate the results of such analysis: at the *DAP* and *FLI1* genes, for example, HS treatment appeared to have little, if any, effect on transcription in the 5′ region, while activity progressively decreased in the rest of the gene (Figure 1A). These and other similar observations (Figure S2A) were confirmed by qPCR analysis (Figure S2B). In these follow-up experiments, we also noticed that transcription typically recovered between 1 and 3 h after cessation of HS, depending on gene length.

Metagene analysis confirmed that this effect on transcription was general; while transcription near the 5′-end of genes was largely unperturbed in response to HS, a progressive decrease in activity was observed toward the 3′-end of genes (Figure 1B). This pattern was most easily observed in longer genes. Furthermore, coverage plots of the same data, obtained by aligning genes at their transcription start sites (TSSs), indicated that HS and control profiles typically begin to diverge only 10–30 kb downstream of the TSS (Figure 1C), well beyond the promotor-proximal pause site. This was also observed in the individual gene examples (Figures 1A and S2A).

To study the behavior of the genes downregulated by HS, we also performed differential gene expression analysis, which resulted in the identification of 2,324 downregulated genes and 1,622 upregulated genes (2-fold change; FDR 0.01) (Table S1). The two subsets were then used for travel ratio analysis (Figure 1D), calculated by dividing the reads in the promoter-proximal region by the reads in the rest of the gene (Reppas et al., 2006). Downregulated genes showed a clear shift toward higher ratios, reflecting the reduction of activity in the gene body. By contrast, upregulated genes exhibited a shift in the opposite direction, consistent with the increased transcription. As suggested by the data in Figure 1, longer genes were relatively more affected, with ∼55% of genes longer than 90 kb downregulated this way, while only ∼32% of genes shorter than 15 kb were similarly affected (Figure S2C).

To investigate the generality of the phenomenon of decreased elongation after HS, we also performed a metagene analysis of previously published PRO-seq data from human embryonic kidney 293 (HEK293) cells exposed to mild HS for 90 min (Aprile-Garcia et al., 2019). To focus on differences in nascent RNA production across the entire gene body, we excluded from the analysis the first 500-bp downstream of the TSS, which are dominant in PRO-seq experiments. Interestingly, also in this cell line, there are dramatic HS-induced reductions in transcription activity that occur well beyond the pause release site (Figure S3A). This was also illustrated by single-gene examples, which showed a similar overall transcription pattern in MRC5-VA and HEK293, despite the different techniques used for measuring transcription activity (Figure S3B).

### HS impairs RNAPII processivity

Due to the approach used, we could not initially rule out that we were observing a transcription recovery phase from an earlier initiation- or pause-release inhibition, rather than repression of transcription. We therefore performed a time course experiment. Here, nascent transcription was analyzed in samples exposed either to 30, 60, or 180 min of HS.

**Figure 2:**
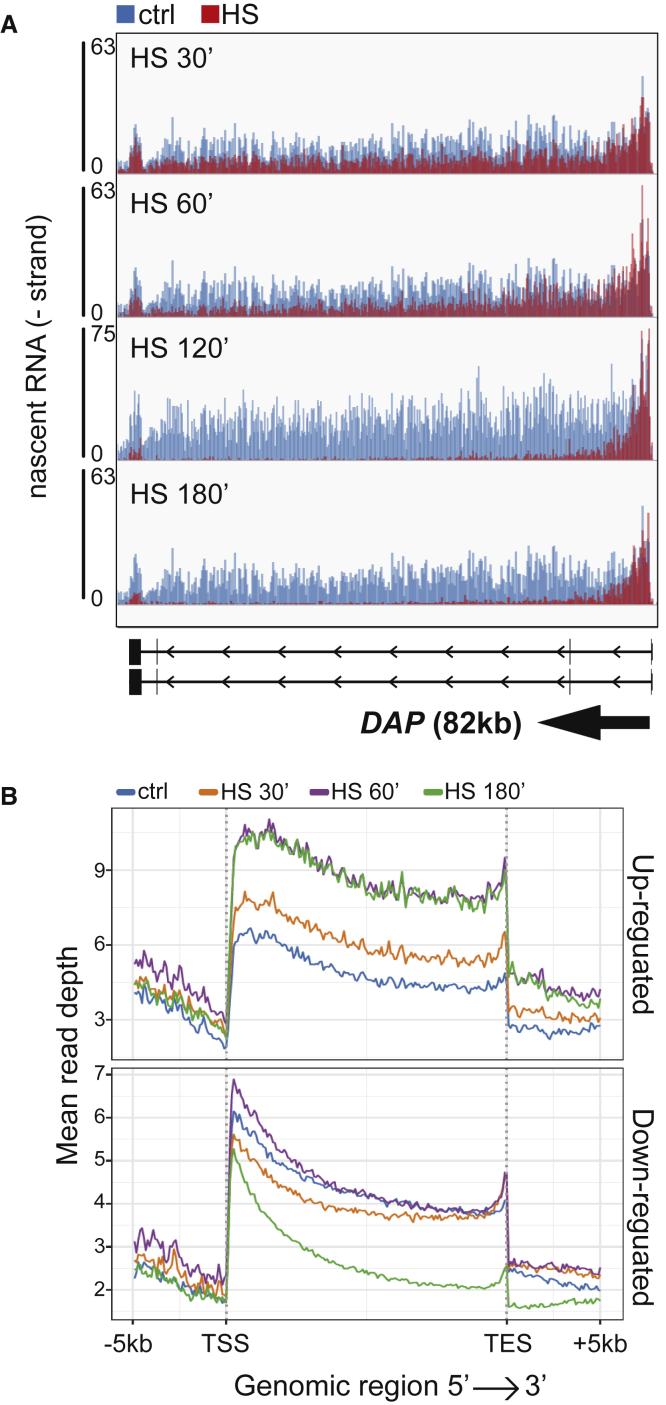
Heat shock impairs transcription processivity (A) IGV genome browser views of TT_chem_-seq for the *DAP* gene in cells exposed to HS for different times. (B) Metagene TT_chem_-seq profiles over time of genes upregulated (upper panel) or downregulated (lower panel) by HS. See also Figure S4.

The *DAP* gene illustrates the results obtained (Figure 2A; see also Figure S4A for more examples). As time progresses, less and less transcription is observed in the downstream regions of the gene, while substantial activity is detected in the 5′-end throughout. Metagene analysis was performed on upregulated and downregulated genes separately. Upregulated genes showed the expected increase in reads throughout the transcription unit already after 30 min (Figure 2B, upper panel). More importantly, downregulated genes were generally only modestly affected with shorter treatment (30 and 60 min) while at the longest treatment (180 min) there was a clear reduction in reads toward the 3′-end (Figure 2B, lower panel), like the results obtained at the 2-h treatment in the previous experiments (Figure 1). It is, however, worth noting that although we do not observe a striking pattern of downregulation over the entire gene unit at earlier time points in the metagene profiles, the single gene examples clearly show that downregulation has already started at 30 and 60 min, but that it has not reached complete shutdown of the gene, or the end of the gene unit. In general, it is only the 3′ end of the downregulated genes that is affected, with activity in this region progressively reduced to a level that then remains largely unchanged between 2- and 3-h treatments.

Interestingly, we also uncovered a small group of long genes, such as *STK39* and *ATXN10*, in which a marked drop of reads is observed with 180-min treatment, and which appear to be the result of a wave of decreased activity originating in the beginning of the gene which then spread over time to the rest of the gene (Figures S4B and S4C). Intriguingly, the region in which the decrease in reads first occurs is again downstream of the promotor-proximal pause site (see Figure S4C), suggesting that this phenomenon is also not explained by inhibition of pause release.

### HS increases the speed of elongation

Previous studies had suggested that HS might not alter elongation speed (Ardehali and Lis, 2009; Mahat et al., 2016). However, considering our results, we now sought to directly measure the elongation rate during HS. For this purpose, we first utilized the CDK9 inhibitor 5,6-dichloro-1-beta-D-ribofuranosylbenzimidazole (DRB) with TT_chem_-seq, in DRB/TT_chem_-seq (Gregersen et al., 2019, 2020). Here, the position of RNAPII in the body of genes is analyzed by TT_chem_-seq at different times after removing DRB to release RNAPII from promoter-proximal areas. The labeling and collection steps were designed to obtain a transcription time course from 0 to 40 min after DRB removal under HS conditions.

**Figure 3:**
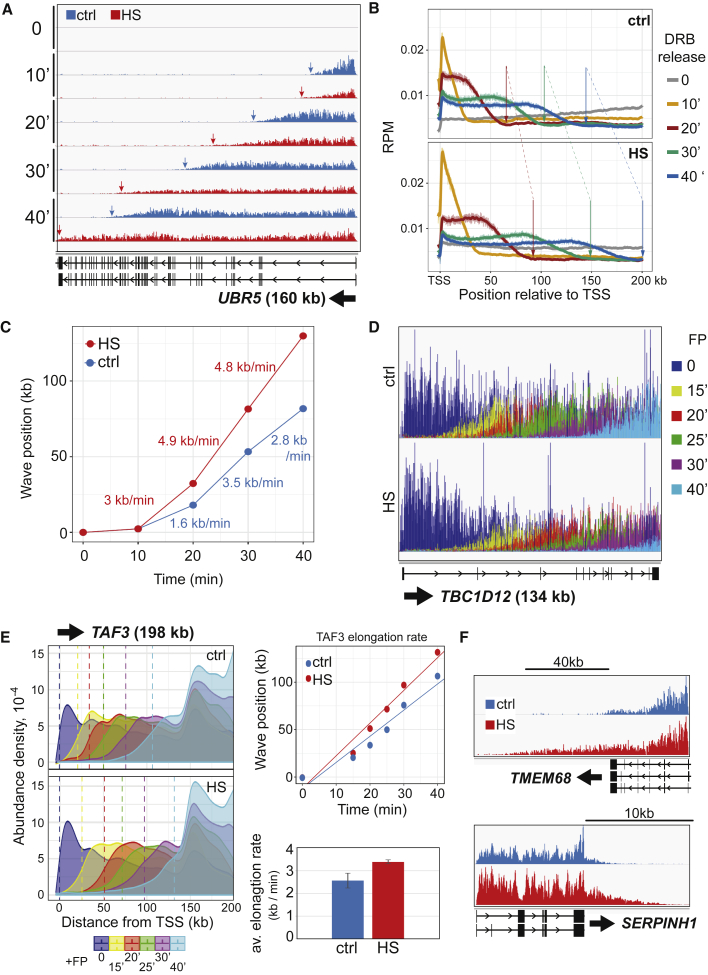
HS increases the speed of transcription elongation (A) IGV genome browser view of DRB/TT_chem_-seq for the *UBR5* gene. Arrows indicate the front of the transcription wave. (B) DRB/TT_chem_-seq metagene profiles. Arrows indicate the front of the transcription wave. (C) Plot of transcription as a function of time after DRB release to calculate elongation rate. The rates between time points are indicated for the two conditions. (D) IGV genome browser view of FP/TT_chem_-seq for *TBC1D12* gene. (E) Left, *TAF3* gene coverage profile as smoothed density from FP/TT_chem_-seq; dashed lines indicate the intersect with the x axis of the midpoint at the rear end of the receding waves. Right top, plot of receding wave position as a function of time after FP addition in the *TAF3* gene. Right bottom, average elongation rate calculated using the *TAF3, MCU*, and *TBC1D12* genes. Error bars indicate ±SD. (F) Readthrough examples; IGV genome browser view of TT_chem_-seq for *TMEM68* and *SERPINH1*. See also Figures S5, S6, and S12.

Surprisingly, single gene profiles and wavefront peak calling analysis clearly showed that RNAPII advances further into genes in HS conditions (Figures 3A, 3B, and S5A). The average transcript elongation rate thus increased from ∼2.1 kb/min to ∼3.4 kb/min after HS, a 60% increase. This was not the result of faster release from pausing as the elongation rates between the different time points were consistently higher and increasing with time (i.e., further into the gene) (Figure 3C).

The increase in elongation rates was unexpected, especially given that it was also observed in genes that are downregulated by HS. Interestingly, however, alongside the primary observations on elongation rate, we noticed that the downregulated genes appeared to be transcribed at an overall level similar to that of untreated cells for the first 20 min following DRB release and only started showing signs of repression at the 30 min time point (see examples in Figure S5B). Given that these cells had already been subjected to HS for 2 h prior to DRB release, this shows the establishment of repression is transcription dependent, e.g., that a factor(s) required for RNAPII to reach the end of genes is being increasingly “depleted” by transcription activity in HS under normal conditions, but not in DRB where there is little or no transcription. Importantly, this likely explains how evidence of transcription not proceeding very far into genes after HS could be obtained by TT_chem_-seq (Figure 1), only for RNAPII apparently running “further” into genes after HS in the DRB/TT_chem_-seq experiments (Figure 3). As discussed later, this observation also provides important evidence for the underlying mechanism.

Given the unexpected nature of these findings, we also investigated the effect of HS by using the kinase inhibitor flavopiridol (FP) combined with TT_chem_-seq. In this approach (Jonkers et al., 2014), addition of FP prevents new transcription complexes from being released from promoter-proximal areas but allows already elongating polymerases to proceed. Over time, this leads to regions of increasing size without transcription activity as elongating polymerases vacate them. Briefly, after exposing cells to HS for 2 h so that repression was established, FP was added and TT_chem_-seq performed at five consecutive time points in HS conditions. For obvious reasons, it was not possible to follow the dynamics of RNAPII clearance in the genes that were most severely downregulated by HS because the region remaining actively transcribed in these genes was typically too short for the earliest time point analyzed (see example in Figure S6A, left). However, genes that were only mildly affected by HS and genes insensitive to HS showed markedly faster RNAPII clearance in HS conditions (Figures 3D and S6A, right). Measuring average elongation rates across the genome is very challenging using FP/TT_chem_-seq, but we calculated the rate on three individual genes: *MCU* (2.09 kb/min without HS; 3.45 kb/min during HS), *TAF3* (2.76/3.45) and *TBC1D12* (2.8/3.26), suggesting an increase in average rate from ∼2.6 kb/min to ∼3.4 kb/min during HS (Figure 3E), akin to what was observed by DRB/TT_chem_-seq.

Moreover, to obtain a genome-wide view, we aligned all long measurable genes at their TSS and generated an average coverage plot of the first 120 kb (Figure S6B). In this approach, the slope on the posterior side of the transcriptional wave is indicative of the extent of clearance by the elongating RNAPII and therefore attests to its speed. Progressively shallower slopes were observed for increasing time points, indicative of RNAPIIs having increasingly vacated these areas of genes. Tellingly, the posterior side of the transcription wave was markedly shallower for the HS samples at every single time point, indicating more clearance and thus faster elongation speed, across the genome (Figure S6B).

Fast elongation often results in increased readthrough beyond the normal transcription end site (TES) (McDowell et al., 1994; Fong et al., 2015; Gregersen et al., 2019). Interestingly, terminator readthrough had actually already been reported in mouse cells exposed to HS (Vilborg et al., 2017; Cardiello et al., 2018). Given its relevance as an indicator of increased elongation rates, we investigated readthrough by measuring the relative transcription levels in regions 20-kb downstream of the TES. HS resulted in a clear increase in readthrough, which was more pronounced for upregulated genes (Figures 3F and S6C). Actually, the presence of frequent readthrough almost certainly led to an overestimate of the number of upregulated genes in our initial expression analysis: some of the genes in this category thus appear to represent false positives due to readthrough transcription from an upstream HS gene (Figure S6D).

### Evidence that HS results in widespread usage of cryptic IPA sites

**Figure 4:**
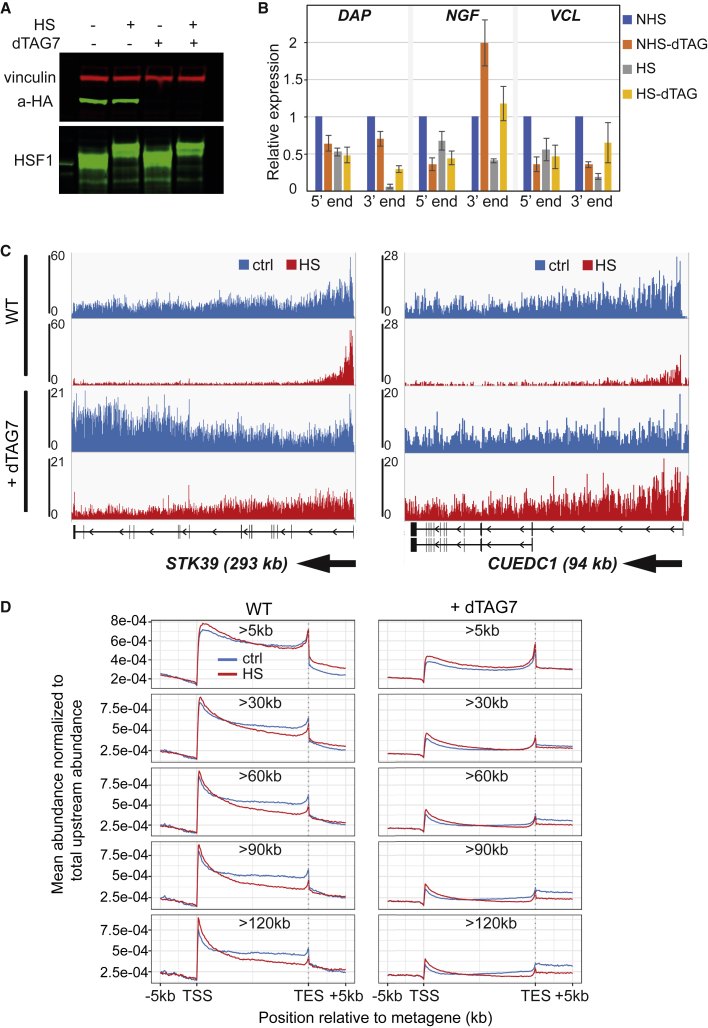
CPSF73 depletion rescues HS downregulation (A) Western blot analysis of CPSF73(dTAG)-HA cells treated with dTAG7 degradation-inducer and HS. Vinculin is used as a loading control and HSF1 as a control for HS treatment. (B) qPCR quantification of nascent RNA near the 5′-end or the 3′-end of the indicated genes, relative to *GAPDH*, normalized to the control. Average of three biological replicates; error bars indicate ±SD. (C) IGV genome browser views of TT_chem_-seq for the *STK39* and *CUEDC1* genes in cells exposed to HS (or not), without (WT) or with CPSF73 depletion (+dTAG7). (D) Metagene TT_chem_-seq profiles. See also Figure S7.

Whether the increase in elongation rate plays a role in the downregulation of genes during HS was unclear. However, if it does, it would appear to not be through the established positive correlation between elongation rate and processivity (Mason and Struhl, 2005; Fong et al., 2015; Liang et al., 2018; Geisberg et al., 2020). One explanation for low processivity in HS would be premature termination brought about by the canonical termination factors acting at cryptic IPA sites. To investigate this possibility, we generated a cell line in which cleavage and polyadenylation specificity factor 73 KDa (CPSF73, also known as CPSF3) can be rapidly depleted using the dTAG system (Nabet et al., 2018; Figures 4A, S7A, and S7B). CPSF73 is required for transcript cleavage at both IPA and 3′-end poly-adenylation sites, and thus for transcriptional termination (Kamieniarz-Gdula and Proudfoot, 2019). We tested the effect of CPSF73 depletion on nascent transcription after HS, initially by RT-qPCR using primers targeting introns at the 5′end and the 3′end of the analyzed genes, respectively. Consistent with other data (Eaton et al., 2020), we observed that CPSF73 depletion resulted in general downregulation of transcription as can be seen from the generally lower signal at the 5′ end of genes. Importantly, however, despite the overall reduction of transcription, we observed a substantial recovery of transcription levels at the 3′-end of downregulated genes after HS (Figure 4B).

Encouraged by these results, we expanded the analysis to the genome-wide level, generating TT_chem_-seq maps upon CPSF73 depletion. Remarkably, depleting this poly(A)-site-specific RNA endonuclease largely reversed the effect of HS on transcription, so that RNAPII could now transcribe to the end of genes even in HS (single gene examples in Figures 4C, S7C, and S7D; genome-wide analysis in Figures 4D). Together, these data strongly indicate an important role for transcriptional termination downstream of premature/cryptic IPA sites in the mechanism underlying transcription downregulation after HS.

### HS transcription and U1 telescripting

Besides CPSF73, several other factors are known to affect PCPA, including PCF11 and CPSF6 (Li et al., 2015; Kamieniarz-Gdula et al., 2019; So et al., 2019; Wang et al., 2019), RNAPII kinases such as CDK12 and CDK13 (Dubbury et al., 2018; Krajewska et al., 2019), SCAF4/SCAF8 (Gregersen et al., 2019), and U1 snRNP in the context of U1 telescripting (Di et al., 2019). Interestingly, we found that U1 expression is markedly downregulated by HS in MRC5-VA cells (Figure S8A). U1, otherwise best known as a component of the spliceosome (Lerner et al., 1980; Papasaikas and Valcarcel, 2016), serves the additional role of preventing the recognition of cryptic IPA sites by the transcription termination factors, effectively repressing PCPA (Kaida et al., 2010; Di et al., 2019). Its depletion by siRNA, or interference with its binding to the nascent RNA by morpholinos, thus leads to suppression of telescripting, via increased usage of IPA sites and increased short isoform production (Berg et al., 2012; Oh et al., 2017; So et al., 2019). Given that our initial results were in effect already clear evidence for inhibition of telescripting (i.e., inhibition of the ability to transcribe “far”), we therefore investigated whether there was a connection between HS-induced termination and this U1 mechanism.

Several genomic features correlate with sensitivity to the disruption of U1-telescripting and more broadly to the likelihood of PCPA occurrence, including gene length (Oh et al., 2017; Krajewska et al., 2019; Wang et al., 2019). We first found that HS-downregulated genes were indeed generally longer (Figure S8B, left). Actually, after HS, the shorter the gene, the more likely it was that its transcription level increased, and conversely, the longer the gene, the more likely its transcription level decreased (Figure S8B, right). Importantly, other features were also consistent with the idea that HS downregulation is due to the inhibition of U1 telescripting: HS-downregulated genes have longer introns, high frequency of IPA sites, low GC content, and low U1/PAS ratio (Figure S8C; see STAR Methods for details) (Oh et al., 2017; Krajewska et al., 2019; Wang et al., 2019). By contrast, HS-induced genes and unchanged genes do not show these features, lowering their sensitivity to disruption of telescripting. In fact, the classical HS genes are typically short and intron-less, which may explain how they escape this repression mechanism.

**Figure 5:**
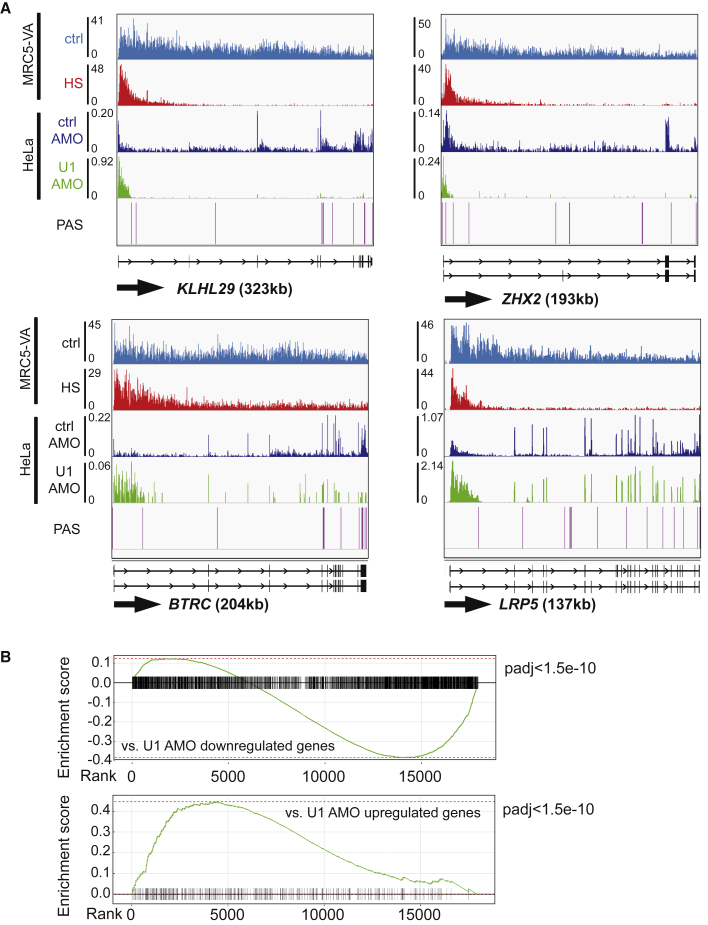
HS mimics telescripting disruption (A) IGV genome browser views of TT_chem_-seq for the *KLHL29*, *ZHX2*, *BTRC*, and *LRP5* genes in MRC5-VA cells, aligned with nascent RNA from HeLa cells treated either with ctrl- or U1-AMO (So et al., 2019) and polyadenylation sites (PASs) from the PolyA_DB database (Wang et al., 2018). (B) Top, GSEA analysis showing the degree of overlap between the HS treatment in MRC5-VA cells, with genes ranked from most upregulated (far left) to most downregulated (far right), and genes resulting in PCPA after U1-AMO treatment in HeLa cells. Bottom, as above but compared with genes upregulated after treatment with U1-AMO in HeLa cells. The green lines represent a running enrichment score. The p values adjusted (padj) are shown. See also Figure S8.

Most significantly, however, we observed that the pattern and manner of gene downregulation in MRC5-VA during HS is strikingly similar to that previously described in HeLa cells in which U1 function was artificially disrupted using U1 morpholinos (U1 AMO, U1 antisense morpholino oligonucleotide) (So et al., 2019), with high levels of transcription in the 5′ region of the genes, but similar, dramatic reductions further into the gene (Figure 5A). The correlation between HS downregulation and disruption of U1 telescripting was confirmed at the global level by gene set enrichment analysis (GSEA) (Subramanian et al., 2005) using a list of genes resulting in PCPA following U1 AMO treatment (Oh et al., 2017; Figure 5B, top panel). Here, genes from the HS dataset were ranked by their level of differential gene expression and compared with genes showing PCPA after exposure to U1 AMO, with the enrichment score showing a strong correlation between the downregulated gene sets (ES −0.38, padj < 1.5e−10). A similar overlap was observed between upregulated genes (Figure 5B, bottom panel) (ES 0.45, padj < 1.5e−10), further supporting U1 involvement in the HS response.

### The genes downregulated by HS express new, shorter mRNAs

The data presented so far were on nascent RNAPII transcription. Now, to gain insight into the fate of the RNA produced during HS, and to further investigate the involvement of telescripting in determining isoform production, we examined the generation of new mature mRNAs, using *neo*-mRNA-seq. In this approach, after exposing cells to elevated temperature to establish downregulation of genes, 4SU was added for an additional period of RNA labeling under continued HS conditions. By subsequently isolating the polyadenylated fraction of the 4SU-labeled RNA, we could enrich for new, mature mRNAs produced during a time in which HS-mediated gene downregulation was being established.

**Figure 6:**
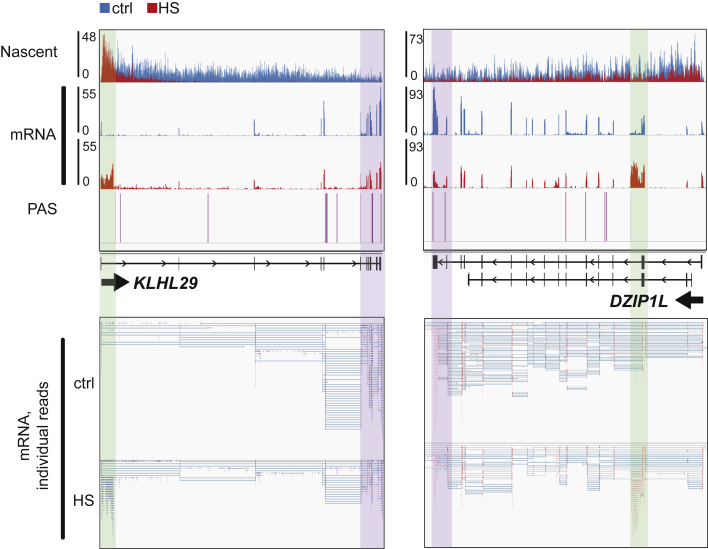
HS induces widespread premature termination IGV genome browser view of TT_chem_-seq, *neo*-mRNA-seq, and polyadenylation sites (PASs) (Wang et al., 2018) for the *KLHL29* and *DZIP1L* genes. For the individual mRNA reads (bottom panels), blue indicates the (+) strand and red the (−) strand. See also Figure S9.

Innumerable genes showed a pattern indicative of shorter mRNA isoform production during HS, correlating well with the profile of the nascent RNA and position of the cryptic IPA sites (Figures 6 and S9A). This shows that the nascent transcripts detected by TT_chem_-seq generate stable mRNAs. Tellingly, while purification of poly(A)-tagged mRNA normally results in overrepresentation of 3′, poly(A)-proximal exons because of random RNA breaks occurring during sample preparation, we instead observed a clear shift toward enrichment of 5′-end polyadenylated gene fragments after HS. Although these shorter mRNA isoforms are typically not annotated, their bona fide nature was demonstrated by a dramatic increase of reads in the 5′ proximal exons (as well as in portions of the adjacent introns, marked by green background) and, more importantly, by a relative lack of splicing to exons downstream of these enriched regions. This correlated with a decrease in signal from canonical exons further downstream (Figures 6 and S9A, marked by purple background). Importantly, the putative 3′-ends of these shorter isoforms align nicely with previously mapped IPA sites (see PASs, Figures 6 and S9A) from the PolyA_DB database (Wang et al., 2018), as expected from inhibition of U1 telescripting. Together, these features point to widespread premature transcript termination being triggered through the activation of cryptic IPA sites after HS, and thus the pervasive production of new mRNA species with the potential to encode new protein/peptide species. Key results were confirmed by qPCR analysis of newly synthesized mRNA, quantifying the relative abundance of the putative proximal terminal exon relative to the canonical distal terminal exon during HS (Figure S9B). We note that qPCR analysis performed on total mRNA, without selecting for the mRNAs produced specifically during the HS treatment, led to similar conclusions (Figure S9C).

**Figure 7:**
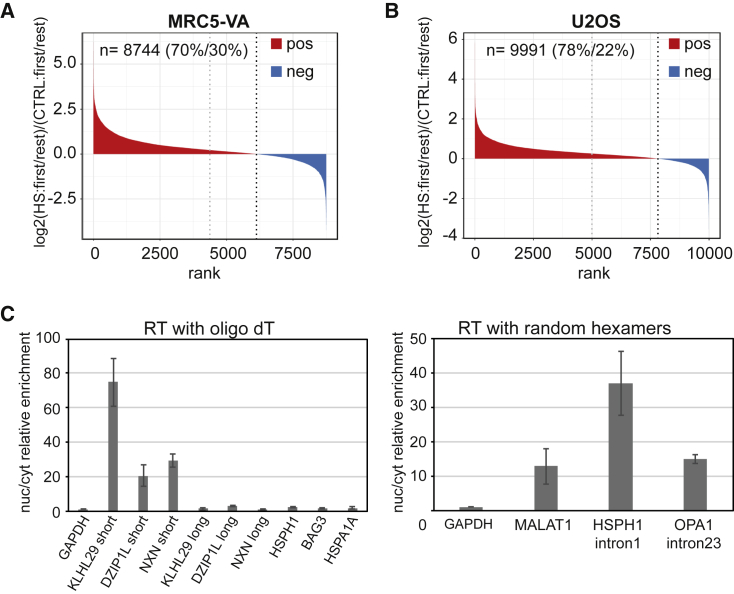
HS induces widespread premature termination (A) First exon enrichment analysis plot of *neo*-mRNA-seq data from MRC5-VA cells. (B) First exon enrichment analysis plot of RNA-seq data from U2OS cells. (C) Left, RT-qPCR quantification in the nuclear fraction relative to the cytoplasm, of the short and long isoforms of *KLHL29*, *DIZIP1L*, and *NXN* mRNA and other canonical mRNAs (RT with oligoT), normalized to *GAPDH* (set to 1). Right, similar data for the stable non-coding RNA, MALAT1, and the nascent transcripts from *HSPH1* and *OPA1* (RT with random hexamers) for comparison. Average of three biological replicates, error bars indicate ± SD. See also Figures S10 and S11.

If premature termination generally occurs across the genome during HS to generate shorter stable mRNAs, a relative increase in reads of the first annotated exon compared with the other exons in the gene should be detected, across the *neo*-mRNA-seq dataset. Indeed, in a “first-exon enrichment analysis” no less than 70% of all transcripts showed a relative enrichment of the first exon after HS (Figure 7A). Tellingly, a similar pattern of expression, characterized by a general and strong enrichment in expression of 5′ proximal regions, can also be observed without selecting for newly made transcripts, in a previously published RNA-seq dataset of U2OS cells exposed to HS (Seifert et al., 2015; Figures 7B and S10).

We note that although thousands of stable mRNAs are generated, a fraction of the premature termination events caused by HS will potentially produce unstable RNAs that are degraded by the exosome, which (although a consequence of premature termination) would result in an even reduction of all the exons of certain genes. Examples of genes that may be representative of this category are shown in Figure S9D.

### mRNA translation?

To investigate whether the new, unannotated mRNA transcripts might be translated into novel proteins, we performed whole-proteome mass spectrometry analysis. For this purpose, we generated a virtual library of open reading frames designed to detect potential protein products (Table S2; Figure S11; see STAR Methods for details). The theoretical tryptic peptides produced from this library were then used to search data from mass spec analysis of HS-treated cells and their controls. Such analysis uncovered few, if any, novel polypeptides from this library and those detected were present at similar level in HS and control conditions (Table S3). This suggests that the dramatic transcriptome-wide restructuring observed after HS does not translate into a new proteome to a substantial degree. One possible explanation for this would be if the novel mRNA transcripts remain in the nucleus. Indeed, short HS-induced transcripts such as those from *KLHL29*, *DZIP1L*, and *NXN* were markedly enriched in the nucleus, in contrast to the normal, “long” forms expressed from the same genes and other mature mRNAs tested (Figure 7C, left) but akin to the non-coding RNA *MALAT1* and nascent RNA expressed from *HSPH1* and *OPA1* (Figure 7, right).

Together, these results indicate that HS induces a dramatic reconfiguration of the human transcriptome, with new, short, typically unannotated transcripts being produced. These do not appear to be significantly translated but may instead remain predominantly nuclear.

## Discussion

HS affects the expression of a surprisingly large fraction of human genes. A limited number of these are dramatically and rapidly upregulated by HSF1-mediated gene activation. However, most genes are downregulated, and the underlying mechanism has remained unclear. In this report, we show that HS results in a general increase in transcript elongation rates, but also a profound failure of RNAPII to transcribe the entire gene, so that much fewer full-length transcripts are produced. Transcripts across the genome are prematurely terminated at cryptic IPA sites, likely through inhibition of U1 telescripting, providing the first example of widespread activation/usage of cryptic IPA sites in response to a physiological stimulus.

### Premature transcription termination is an integral part of heat-shock-mediated gene repression

Global transcription downregulation is a conserved but poorly understood feature of the HS response (Jamrich et al., 1977; Gasch et al., 2000; Mahat et al., 2016). Somewhat surprisingly, this dramatic change in transcription does not correlate with a reorganization of chromatin structure (Teves and Henikoff, 2011; Ray et al., 2019). Moreover, it is clear that at the vast majority of genes, transcription starts normally but progression through the gene body is prevented (Allen et al., 2004; Hieda et al., 2005; Mariner et al., 2008; Teves and Henikoff, 2011; Mahat et al., 2016; Vihervaara et al., 2017; Aprile-Garcia et al., 2019; Rawat et al., 2021). The prevalent current model describes this phenomenon as an effect of pause-release inhibition. However, while previous studies were highly effective in analyzing the initial phases of the transcriptional cycle, the genome-wide approaches used (typically PRO-seq and ChIP-seq) are arguably less well suited for investigating events occurring during later stages such as elongation and termination. Using TT_Chem_-seq analysis in CPSF73-depleted cells, we found that the impairment observed in RNAPII processivity is caused by premature transcript termination by the canonical termination machinery. *neo*-mRNA-seq analysis of mRNA synthesized during HS showed that this leads to the production of short, typically unannotated mRNAs, resulting from the activation of cryptic IPA sites. This provides an unexpected, but compelling explanation for HS-induced downregulation of genes.

In conjunction with the previous data on pause release, our results support a model in which multiple mechanisms concur to achieve transcription repression during HS, and that among them, defective processivity and premature termination play a principal role, certainly during prolonged HS where the substantial downregulation of gene expression is observed.

### HS elicits an increase in transcription elongation rate

Previous attempts to determine the effect of HS on elongation rate led to conflicting conclusions in different organisms (Ardehali and Lis, 2009; Miguel et al., 2013; Mahat et al., 2016). Through direct measurement of elongation speed using two complementary approaches, we now demonstrate that elongation speed increases significantly during HS in human cells. Such an increase might either be a direct consequence of faster biochemical reaction rates, the result of regulation by co-factors, or both. Given that RNA polymerases move by Brownian motion (Bar-Nahum et al., 2005), it might be expected to transcribe faster at higher temperature unless critical protein domains required for nucleotide addition and other fundamental polymerase functions begin to unfold and loose optimal function. We note that transcription elongation experiments with highly purified mammalian RNAPII alone failed to show a noteworthy difference in elongation rates between 37°C and 43°C *in vitro* (Figure S12). Conversely, factors such as EF1A1 and B2 RNA affect elongation by RNAPII during HS (Vera et al., 2014; Zovoilis et al., 2016), suggesting that RNAPII elongation rates might be elevated through the activation of such co-factors. The basis for the marked increase in cellular elongation rates after HS requires further study.

In general, the elongation phase of transcription can be described in terms of speed and processivity. These features are tightly linked, with higher elongation rates correlating with higher processivity (Mason and Struhl, 2005; Liang et al., 2018; Geisberg et al., 2020) rather than decreased processivity as observed here. At the same time, however, slow elongation also typically results in increased exon inclusion during co-transcriptional splicing (de la Mata et al., 2003), whereas fast elongation generally results in exon exclusion (Saldi et al., 2016). The kinetic coupling model (Naftelberg et al., 2015) thus posits that slow elongation enlarges (while fast elongation reduces) the time window for recognition of upstream splice sites, thereby increasing (or decreasing) inclusion of alternative exons during co-transcriptional mRNA splicing. Intriguingly, as illustrated by the U1 morpholino experiments (Venters et al., 2019), telescripting likewise requires efficient recognition of the 5′ splice site, by U1. Akin to what is proposed in the kinetic coupling model, it is thus possible that the very high elongation rate observed during HS results in less efficient recognition of the (fast-moving) splice sites or the cryptic IPA sites themselves by the RNAPII-associated U1 snRNP, so that premature termination at cryptic IPA sites becomes more prevalent. During HS, increased elongation rates thus correlate with “decreased” processivity, but arguably in an explicable manner.

Interestingly, it seems possible that, during HS, increased elongation rates might at the same time be responsible for premature termination at some genes and transcription readthrough of canonical terminators at others. Such readthrough was previously described in mouse cells (Vilborg et al., 2017; Cardiello et al., 2018) but is detectable in our system as well; it is particularly evident in genes that are upregulated by HS. Intriguingly, the dramatic increase in elongation rate observed during HS raises the possibility that the readthrough “downstream of genes (DOGs)” transcripts reported by Steitz and co-workers during osmotic stress (Vilborg et al., 2015; Rosa-Mercado et al., 2021) might also, at least partly, be a result of increased elongation rates under these conditions. This should be investigated.

### Premature termination regulation and U1-telescripting

Most human genes contain cryptic PASs in introns and these elements represent a constant, potential threat to normal gene expression (Tian and Manley, 2017; Auboeuf, 2018). Their recognition by the termination machinery is prevented by telescripting through a splicing-independent function of U1 snRNP (Di et al., 2019), or by the SCAF4/8 anti-terminator proteins (Gregersen et al., 2019). Other factors have been shown to participate in cryptic IPA site selection, either by stimulating (PCF11) or suppressing PCPA events (CPSF6, CDK12, and CDK13), but it is not yet clear whether they function independently or in the context of U1 telescripting or SCAF proteins (Li et al., 2015; Dubbury et al., 2018; Kamieniarz-Gdula et al., 2019; Krajewska et al., 2019; So et al., 2019; Wang et al., 2019).

The first ground-breaking evidence for the existence of telescripting was obtained by using U1-AMOs to compete for U1’s binding to the 5′ splicing site in nascent RNA (Kaida et al., 2010). Such treatment not only compromised mRNA splicing but also resulted in widespread premature termination. Although it has long been clear that a low level of IPA normally occur in most genes, and that a handful of specific gene expression events are regulated via alternative polyadenylation (Vorlová et al., 2011; Oh et al., 2017; Kamieniarz-Gdula et al., 2019; Wang et al., 2019), it has not previously been clear if IPA, on a genome-wide scale, can be triggered in response to a physiological stimulus. It has thus remained unclear, for example, why cells have retained IPA sites despite their obvious harmful potential. Because long genes tend to rely more on U1-telescripting, it was speculated that a general, transient switch to PCPA isoforms might be favorable in acute responses, to decrease competition for RNA processing factors (Oh et al., 2017). In line with a key role for inhibition of telescripting in determining HS-induced transcription shutdown, we find that long genes are indeed particularly affected. Several other features that characterize genes inhibited by telescripting are observed in HS-repressed genes as well.

It is important to stress that several pathways might cooperate to inhibit telescripting during HS. We initially pursued the line of investigation described here partly based on the discovery that the U1 gene is strongly repressed during HS. Frustratingly, our attempts to reverse the effect of HS by artificially overexpressing U1 were hampered for technical reasons. However, it has also been reported, for example, that SRSF10 is dephosphorylated during HS, and that this can prevent U1 snRNP binding to nascent RNA, resulting in splicing defects (Shin et al., 2004; Shi and Manley, 2007). It seems likely that a reduction of available U1 snRNPs activity this way might affect PCPA events as well. Even without reducing the total amount of U1 snRNPs, a transient imbalance between overall transcription levels and available U1 snRNPs can be sufficient to trigger PCPA events (Berg et al., 2012). This situation would likely apply to HS where induced genes reach very high level of transcription and thus might “deplete” U1 levels. Indeed, an U1 snRNP imbalance being generated by inducing transcription would be in agreement with the delay in establishing repression observed when transcription restarts after DRB release even though cells were already in HS (Figure S5B). Finally, as described above, the elevated rate of elongation during HS might itself impede recognition by U1 of 5′ splice sites, and the cryptic IPA sites themselves, and thus result in an inability to suppress the recognition of cryptic IPA sites, further helping to trigger premature termination.

The HS-induced genes escape disruption of telescripting for several reasons. First, most of the classical HS genes are short and intron-less, and more generally, genes induced by HS present genomic features that confer higher resistance to U1 depletion such as fewer and shorter introns. It is, of course, also possible that U1 is recruited to HS genes to prioritize their correct transcription and processing, akin to what happens to mRNA splicing which changes during HS as well (Biamonti and Caceres, 2009; Shalgi et al., 2014; Hussong et al., 2017).

### Why terminate genes prematurely?

While the physiological importance of the mechanism outlined here is difficult to establish due to the inordinately large number of genes affected, it is an obvious possibility that a global shift to expressing short mRNA transcripts, in response to an ancient physiological stress, might help explain why cryptic PASs in introns have been retained during evolution. So, why might such a mechanism have evolved?

First, premature termination at the 5′-ends of genes that are not required for the stress response will release more polymerases into the free RNAPII pool as less time is spent transcribing them, which in turn would enable elevated levels of transcription of the typically very short HS genes that need to be highly active for supporting survival during heat stress. Indeed, simple computational modeling of transcription based on our previous work (Ehrensberger et al., 2013; Tufegdžić Vidaković et al., 2020) indicates that the enlarged RNAPII pool resulting from premature termination at thousands of genes across the genome would “in itself” (i.e., even without HSF-activated transcription) allow unaffected (short) genes to be upregulated (see Video S1). As an aside, we note that the upregulation of some genes after U1 morpholino treatment (Venters et al., 2019) might be governed by this mechanism as well; i.e., these genes may be upregulated due to the increased RNAPII pool. In support of this idea, genes upregulated by HS and morpholino treatment significantly overlap (padj < 1.5e−10), despite their (at first glance) very different manner of induction.

Video S1. Animation of computational modeling of transcription of short (5 kb), medium (63 kb), and long (100 kb) model genes based on our previous work (Ehrensberger et al., 2013; Tufegdzic Vidakovic et al., 2020) HS at time = 0 changes transcription dynamics and establishes a new equilibrium with higher-level transcription of the now shorter, model transcription units. See STAR Methods for details. Related to Figures 1, 2, 3, 4, 5, 6, and 7 and discussion

Second, selectively inhibiting the expression of long mRNAs, responsible for the synthesis of large proteins, might ensure that the protein chaperone system is not overwhelmed during HS. Finally, given that the new, short mRNAs appear to accumulate in the nucleus and not support productive protein synthesis, it is tempting to speculate that some of them might have evolved to support HS survival, for example, by acting as lncRNAs or other stable RNAs, allowing more rapid transcription recovery. The fact that genome-wide use of cryptic PASs in introns can support such a multipronged stress response might even explain why such “deleterious” sites have evolved and/or been retained during evolution.

### Limitations of the study

This study provides evidence that the global transcription shutdown induced in the HS response is caused by widespread activation of cryptic IPA sites in introns, resulting in premature termination. This per definition constitutes inhibition of telescripting. However, although we detected repression of U1 snRNA transcription during HS and all features of the HS-induced process also resemble inhibition of U1 telescripting, we do not provide direct evidence that the disruption of telescripting we observed is mediated by regulation of U1 activity. To investigate the importance of U1 for the mechanism, we attempted to either overexpress U1 snRNA by transient transfection of plasmids carrying the gene, or by expressing it from an inducible promoter. However, these approaches failed to yield meaningful results. First, transient transfection of plasmids carrying the U1 snRNA gene is cell lethal, and second, U1 appears to require its own promoter to start transcription at the correct start site and produce a functional snRNA. U1 is generally very abundant and stable, so it is also possible that its activity after HS is regulated by other means than by abundance.

Finally, the precise biological function of the global transcription shutdown described here remains unresolved. As described in the discussion, we can only speculate on its overall effect on, and importance for, the HS response. A reversal of transcription shutdown, of literally thousands of genes, would be required to fully characterize and understand this feature of the HSR. Such reversal is not currently possible.

## STAR★Methods

### Key resources table

**Table:** 

REAGENT or RESOURCE	SOURCE	IDENTIFIER
**Antibodies**		

Polyclonal to HSF1	Enzo Life Sciences	Cat# ADI-SPA-901 RRID:AB_10616511
Monoclonal to Vinculin	Sigma-Aldrich	Cat# V9131 RRID:AB_477629
Polyclonal to CPSF73	Bethyl	Cat# A301-090A RRID: AB_873009
Monoclonal to HA	Abcam	Cat# ab236632 RRID:AB_2864361
Anti-Mouse secondary antibody (HRP)	Santa Cruz	Cat# sc-516102 RRID:AB_2687626
Anti-Rabbit secondary antibody (HRP)	Jackson Immunoresearch	Cat# 711-035-152 RRID:AB_10015282
IRDye 680RD Donkey anti-Mouse secondary antibody	LI-COR Biosciences	Cat# 926-68072 RRID:AB_10953628
IRDye 800CW Goat anti-Rabbit secondary antibody	LI-COR Biosciences	Cat# 926-32211 RRID:AB_621843
Monoclonal to CTD repeat RNAPII (8WG16)	The Francis Crick Institute Core facility	N/A

**Chemicals, Peptides, and Recombinant Proteins**		

4-thiouridine	Glentham Life Sciences	Cat# GN6085
4-thiouracil	Sigma-Aldrich	Cat# 440736
MTSEA biotin-XXlinker ((MTSEA Biotincacap; 2-((6-((6-((biotinoyl)amino)hexanoyl)amino)hexanoyl)amino)ethylmethanethiosulfonate))	Biotium	Cat# BT90066
DRB (5,6-dichloro-1-β-D-ribofuranosylbenzimidazole)	Sigma-Aldrich	Cat# D1916
Flavopiridol hydrochloride hydrate	Sigma-Aldrich	Cat# F3055
Polyethylenimine	Polysciences, Inc.	Cat# 23966-1
Hexadimethrine bromide	Sigma-Aldrich	Cat# H9268
dTAG7	Tocris, Bio-Techne Ltd	Cat# 6912
Blasticidin	TOKU-E	Cat# B007-20ml
Puromycin	Gibco™	Cat# A1113803
Crystal violet	Sigma-Aldrich	Cat# C3886

**Critical Commercial Assays**		

RNeasy mini kit	QIAGEN	Cat# 74106
RNA minElute clean-up kit	QIAGEN	Cat# 74204
PureLink RNA Mini kit	Thermo Fisher Scientific	Cat# 12183020
μMACS Streptavidin Kit	Miltenyi Biotec	Cat# 130-074-101
Taqman Reverse Transcriptase Reagents	Thermo Fisher Scientific	Cat# N8080234
KAPA RNA HyperPrep kit	Roche	Cat# 08098107702
KAPA mRNA HyperPrep kit	Roche	Cat# 08098123702

**Deposited Data**		

Raw FASTQ files and unscaled bigwig files available at NCBI's Gene Expression Omnibusunder	This study	GEO: GSE165368
Sequencing data are available under GEO number GSE165368	This study	GEO: GSE165368
Images available at Mendeley	This study	https://doi.org/10.17632/x7hcmjjpj7.1

**Experimental Models: Cell Lines**		

HEK293T	The Francis Crick Institute Cell Services	N/A
MRC5VA	The Francis Crick Institute Cell Services	N/A
MRC5VA CPSF73 (dTAG)	This study	N/A
MRC5VA shHSF1	This study	N/A
MRC5VA shCtrl	This study	N/A

**Experimental Models: Organisms/Strains**		

*S. cerevisiae* (strain BY4741, MATa, his3D1, leu2D0, met15D0, ura3D0)	Euroscarf	BY4741(Y00000)

**Oligonucleotides**		

All oligonucleotides used in this study are listed in Table S4	This paper	N/A

**Recombinant DNA**		

pLKO.1 shHSF1 TRCN0000007480	GE healthcare Life Sciences	Cat# RHS3979-201739753
pLKO.1 shCtrl	(Williamson et al., 2017)	N/A
pLP1 RRE (GAG POL)	Kind gift from Simon Boulton	N/A
pLP2 RVE (REV)	As above	N/A
pLP/VSVG (VSVG)	As above	N/A
GW223-pX330A-sgX-sgPITCh	Kind gift from Andreas Mayer	N/A

**Software and Algorithms**		

Cutadapt v1.9.1	(Martin, 2011)	https://cutadapt.readthedocs.io/en/stable/index.html
STAR v2.5.2a	(Dobin et al., 2013)	https://github.com/alexdobin/STAR
DESeq2	(Love et al., 2014)	https://bioconductor.org/packages/release/bioc/html/DESeq2.html
GenomicAlignments	(Lawrence et al., 2013)	https://bioconductor.org/packages/release/bioc/html/GenomicAlignments.html
Picard v2.1.1	http://broadinstitute.github.io/picard	http://broadinstitute.github.io/picard
BEDtools v2.27	(Quinlan and Hall, 2010)	https://bedtools.readthedocs.io/en/latest/
KentTools	(Kent et al., 2010)	http://hgdownload.soe.ucsc.edu/admin/exe/
deepTools v2.5.3	(Ramírez et al., 2014)	https://www.deeptools.readthedocs.io/en/develop/#
Trim Galore! v0.4.4	N/A	https://www.bioinformatics.babraham.ac.uk/projects/trim_galore/
SAMtools v1.9	(Li et al., 2009)	http://www.htslib.org/
MaxQuant v1.6.15.0	(Cox and Mann, 2008)	http://www.maxquant.org/

**Other**		

High glucose DMEM	Thermos Fisher Scientific	Cat# 11965118
4-15% TGX gels	BioRad	Cat# 56711084
Complete EDTA-free protease inhibitor cocktail	Sigma-Aldrich	Cat# 05056489001
PhosSTOP™	Sigma-Aldrich	Cat# 04906837001
Nitrocellulose membrane	GE healthcare Life Sciences	Cat# 10600002
SuperSignal™ West Pico PLUS ECL reagent	Thermo Fisher Scientific	Cat# 34577
Micro Bio-Spin™ P-30 Gel Columns	BioRad	Cat# 7326223
iTaq™Universal SYBR® Green Supermix	BioRad	Cat# 172-5124
BaseMuncher	Expedeon	Cat# BM0100
AMPureXP beads	Beckman Coulter	Cat# A63881
TRIzol™ Reagent	Thermo Fisher Scientific	Cat# 15596026
Lipofectamine 3000	Thermo Fisher Scientific	Cat# L3000015
Lyticase	Sigma-Aldrich	Cat# L2524
Immobilon-PVDF membrane	Merck Life Science	IPFL00010
Intercept(PBS) Blocking Buffer	LI-COR	Cat# 927-70001
QuickExtract DNA Extraction solution	Epicentre Technologies/Lucigen	Cat# QE09050
ExoSapIT	Applied Biosystem	Cat# 78201.1.ML
Q5 High Fidelity 2X Master Mix	New England BioLabs	Cat# M0492S
Fast Flow Q Sepharose	Cytiva	Cat# 17051001
Dynabeads MyOne Streptavidin T1	Invitrogen	Cat# 65601
Amicon Ultra 100kDa NMWCO	MerckMillipore	Cat# UFC8100

### Resource availability

#### Lead contact

Relevant material and any information required to reanalyze the data reported in this paper are available from the lead contact, Jesper Svejstrup, at jsvejstrup@sund.ku.dk.

#### Materials availability

Cell lines generated in this study are available from the lead contact without restriction.

### Experimental model and subject details

#### Cell lines and culture conditions

HEK293T (human embryonic kidney epithelial, female origin), MRC5-VA (human fetal lung fibroblast, male origin) and derived cell lines were cultured in high glucose DMEM (Thermo fisher scientific) at 37^°^C with 5% CO_2_. Culture media were supplemented with 10% v/v FBS, 100U/ml penicillin and 100mg/ml streptomycin. All cell lines were confirmed to be mycoplasma-free by the Francis Crick Institue Cell Services. For heat shock (HS), cells were subjected to immediate HS by replacing the media with pre-warmed media at 43^°^C and kept in an incubator at 43^°^C for the indicated period of time. All the sequencing experiments were carried out in conditioned medium.

### Method details

#### Generation of stable RNAi cell line

Viral particles were generated by co-transfecting HEK293T cells with 1 μg third-generation lentiviral packaging vectors (pLP1, pLP2 and pLP/VSVG, a kind gift from the Boulton lab (Crick Institute)) and 1 μg lentiviral vectors (pLKO.1) containing either a HSF1 specific shRNA (TCRN0000007480) or a Non-targeting Control shRNA (shCtrl) using polyethylenimine (PEI) (Polysciences, Inc., 23966-1) as a transfection reagent (3:1 PEI/DNA ratio). 48h from transfection, viral particles were collected and used for transduction of MRC5-VA. Hexadimethrine bromide (Sigma-Aldrich, H9268) was used to increase infection efficiency. Cells were selected in 1 μg/ml puromycin and the knockdown efficiency was tested by western blot.

#### Generation of CPSF73 (dTAG) cell line

The CPSF73 gene was tagged with a C-terminal FKBP degron by the MMEJ homology method described by Nabet et al. (2018). CPSF73 homology arms were inserted into the plasmid pCRIS-PITChv2-dTAG-BSD (BRD4) according to the protocol provided by Nabet et al. The gRNA (GGCTGCACAGAGACTGTACG) was inserted into the Cas9 vector GW223-pX330A-sgX-sgPITCh. Both plasmids were a kind gift from the Mayer Lab. The plasmids were transfected into MRC5-VA cells using Lipofectamine 3000 (Invitrogen, ThermoFisher Scientific) and selected with 5ug/ml Blasticidin (Cambridge Bioscience). Single clones were isolated and the genomic DNA prepared using QuickExtract (Epicentre Technologies) and ExoSapIT (Applied Biosystems). The region surrounding the tag insertion site was amplified with primers ATTAGGACCGTGCTGCTGTC and CCTGTAACACCCACGAGGAC using Q5 polymerase (New England bioLabs). Clones with a PCR product of 3036 bp were expanded and analysed by Western blot using either antibodies to HA (ab 236632 Abcam Ltd) or CPSF73 (A301-090 Bethyl Laboratories Inc). Genomic DNA was sequenced to ensure correct insertion of the degradation tag. Homozygous clones were treated with dTAG-7 (Tocris, Bio-Techne Ltd.) at 250nM for 2h and the degradation of CPSF73 confirmed by Western blot.

#### Clonogenic survival assay

shHSF1 stable cells were seeded at 400 cells/well and shCtrl cells were seeded at 200 cells/well into 6-well plates. Cells were exposed to HS for the indicated amount of time and then kept in incubator at 37^o^C for 11 to 13 days.

Colonies were fixed by 4 % (v/v) formaldehyde and stained with a 0.1 % (w/v) crystal violet solution. Crystal violet was extracted with a 10% acetic acid solution and quantified. Colonies from three biological replicates (each seeded into triplicate wells) were used for the quantification.

#### Western blot

For whole cell extracts, cells were resuspended and incubated for 30 minutes at 4°C in lysis buffer (250 U/ml BaseMuncher Benzonase (Expedeon, BM0100), 150 mM NaCl, 20mM TRIS-HCl pH 8, 0.1% (v/v) NP-40, 10% (v/v) Glycerol, 1.5 mM MgCl_2_) supplemented with protease (Sigma-Aldrich, 05056489001) and phosphatase (PhosSTOP™, Sigma-Aldrich, 04906837001) inhibitors. The lysates were spun at 14000 RPM at 4°C for 15 minutes and the supernatant was used for western blot analysis. Proteins were separated on 4%–15% TGX gels (BioRad, 5671084) and transferred either to a nitrocellulose membrane (GE Healthcare Life Sciences, 10600002) or to a PVDF membrane (Merck Life Science, IPFL00010). Membranes were blocked respectively in 5% (w/v) skimmed milk in PBS supplemented with 0.1% (v/v) Tween20 (PBST) or in Intercept (PBS) blocking buffer (LI-COR, 927-70001) for 1 h at room temperature. Incubation with primary antibodies was carried out in PBST or Intercept (PBS) blocking buffer supplemented with 0.1% (v/v) Tween20 overnight at 4°C. Membranes were washed several times in PBST, incubated with either with HRP-conjugated or LI-COR fluorescent dye-conjugated secondary antibody diluted respectively in PBST or Intercept (PBS) blocking buffer supplemented with 0.1% (v/v) Tween20 for 45 min at room temperature and washed several times in PBST. Signal detection was obtained either using SuperSignal West Pico PLUS (Thermo Fsher Scientific, 34577) Substrate ECL reagent or Odyssey CLx imaging system (LI-COR). All antibodies used are listed in the key resources table.

#### RT-qPCR

Total RNA was extracted using RNeasy mini kit (QIAGEN, 74106) following manufacturer instructions including an on-column DNase digestion (QIAGEN, 79254). 1 to 1.5 μg RNA was used for Reverse transcription with TaqMan Reverse Transcription Reagents (Thermo Fisher Scientific, N8080234) according to manufacturer instructions. For the 4SU-labelled mRNA, approximately 30 to 100ng of material were used for the reverse transcription. For detection of nascent RNA, random hexamers were used in the reverse transcription reaction and for the detection of mRNA, oligo dT primers. cDNA was amplified on a CFX384 Touch Real-Time PCR Detector (BioRad 1855485) using iTAq Universal SYBR Green Supermix (BioRad, 172-5124) with the following conditions: 5 min denaturation at 95°C and 39 cycles of 10 s denaturation at 95°C, 10 s annealing at 58°C, and 20 s extensions at 72°C. Primers amplifying GAPDH mRNA were used for normalization purposes. All primer sequences are listed in Table S4.

#### Nucleus-cytoplasm fractionation

10 x 10^6^ cells were exposed to HS for two hours, washed with cold PBS twice and collected. The cells were then resuspended in 200 μl cytoplasmic lysis buffer (10 U RNase inhibitor (Thermo Fisher Scientific, N8080119), 10 mM Tris-HCl pH 7, 150 mM NaCl, 0.15% NP40) and incubated in ice for 5 min. The samples were then layered over 500 μl sucrose buffer freshly made (20 U RNase inhibitor, 10 mM Tris-HCl pH 7, 150 mM NaCl, 25% sucrose) and centrifuged at 16,000 g for 10 min at 4 °C. The supernatant was collected and used for cytoplasmic RNA extraction with RNeasy mini kit (QIAGEN, 74106) after adding 3.5X RLT Buffer (QIAGEN, 79216) and 2.5X ice cold 100% ethanol. The nuclei pellets were washed with 200 μl sucrose buffer and then used for RNA extraction with RNeasy mini kit (QIAGEN, 74106) following manufacturer instructions including an on-column DNase digestion (QIAGEN, 79254).

#### TT_chem_-seq

The TT_chem_-seq was carried out as described in Gregersen et al. (2020) in at least two biological replicates. 8 X10^6^ cells were exposed to HS and the RNA was labelled *in vivo* with 1 mM 4SU (Glentham Life Sciences, GN6085) for 15 minutes prior to the addition of TRIzol (Thermo Fisher Scientific) that was used to stop the reaction at the desired time point. RNA was extracted according to manufacturer instructions.

As a control for equal sample preparation, we spiked-in 4-thiouracile (4TU) labelled RNA from *S. cerevisiae* (strain BY4741, MATa, his3D1, leu2D0, met15D0, ura3D0). The yeast culture was grown overnight in YPD medium, diluted to OD600 of 0.1 and grown to mid-log phase (OD600 of 0.8) and labelled 5 min with 5 mM 4TU (Sigma-Aldrich, 440736). Total RNA was extracted with PureLink RNA Mini kit (Thermo Fisher Scientific, 12183020) following the enzymatic protocol.

100 μg of human 4SU labelled RNA was spiked-in with 1 μg of 4TU-labelled yeast RNA and brought to a total volume of 100 μl with water. The mix was fragmented by adding 20 μl 1M NaOH and incubating in ice for 20 min. 80 μl of 1M Tris-HCl pH 6.8 were added to stop the fragmentation and samples were cleaned up twice with Micro Bio-Spin P30 Gel Columns (BioRad, 7326250) following manufacturer instructions. Biotinylation of 4SU and 4TU residues was carried out in a total volume of 250 μl 10 mM Tris-HCl pH 7.4 and 1 mM EDTA, containing MTSEA biotin-XXlinker (Biotium, BT90066) for 30 min at room temperature in the dark. The RNA was then purified by phenol:chloroform extraction, denatured 10 min at 65°C and added to 200 μl μMACS Streptavidine MicroBeads (Miltenyi Biotec, 130-074-101). After 15 min incubation at room temperature, the mix was loaded to a μColumn in the magnetic field of a μMACS magnetic separator. The beads were washed twice in a buffer containing 100 mM Tris-HCl pH7.4, 10 mM EDTA, 1M NaCl and 01% Tween20. Biotinylated RNA was eluted twice in 100 mM DTT (200 μl final volume) and cleaned up with the RNeasy MinElute kit (QIAGEN, 74204) using 1050 μl 100%ethanol and 750 μl RLT buffer to precipitate RNA <200nt.

#### DRB/TT_chem_-seq

The DRB/TT_chem_-seq was carried out as described in Gregersen et al. (2020) in two biological replicates. One 150 mM dish was used for each sample, except the samples not released from DRB which required two 150 mM dishes. Cells were incubated in 100 μM DRB (Sigma-Aldrich, D1916) for 1,5 hours. For the HS treatment, the medium in the cells was replaced with medium pre-warmed at 43°C supplemented with 100 μM DRB and the incubation was continued in a 43°C incubator for additional 2 hours. The cells were then washed twice in PBS and fresh medium at 43°C was added to restart transcription. 4SU labelling was carried out for 10 min prior to the addition to TRIzol to stop the reaction at the desired time point between 10 and 40 min after DRB release. Samples were then processed following the TT_chem_-seq protocol.

#### FP/TT_chem_-seq

Cells were kept at 43°C for 2h, the untreated samples (not treated with FP) were labelled with 4SU for 10 min and collected in TRIzol exactly after 2 hours of HS. The remaining samples were kept at 43°C, FP (Sigma-Aldrich, F3055) was added to the cells to a final concentration of 300nM and the 10 min 4SU labelling was timed to obtain five time points between 15 and 40 minutes after FP addition. The cells were disrupted in TRIzol at the desired time point and the samples were then processed following the TT_chem_-seq protocol.

#### 4SU/mRNA-seq

Cells were exposed to HS for 1h and 45 min, RNA was labelled in vivo with 0.7 mM 4SU for 90 min in HS conditions. The cells were harvested by scraping in pre-warmed PBS and span down. The pellets were then snap-frozen in liquid nitrogen and quickly defrosted for RNA extraction with RNeasy mini kit (QIAGEN, 79254) following manufacturer instructions including an on-column DNase digestion (QIAGEN, 79254). 100 μg of 4SU labelled RNA were used for the biotinylation reaction that was carried out in a total volume of 250 μl 10 mM Tris-HCl pH 7.4 and 1 mM EDTA, containing MTSEA biotin-XXlinker (Biotium, BT90066) for 1h at room temperature in the dark. The purification and pull-out of biotinylated RNA was carried out as in the TT_chem_-seq protocol. The material was either used for RT-qPCR or library preparation.

#### Library preparation

For all the TT_chem_-seq experiments and its derivatives (DRB/ and FP/TT_chem_-seq), 30 to 100 ng of 4SU/4TU labelled RNA were used for library preparation with the KAPA RNA HyperPrep kit (Roche, 08098107702) following manufacturer instructions. The fragmentation step was omitted and the RNA, resuspended in FPE Buffer, was denatured at 65°C for 5 min. The libraries of the DRB/TT_chem_-seq and the FP/TT_chem_-seq experiments were prepared with modifications as previously described (Tufegdzic Vidakovic et al., 2020). Briefly, the two SPRI bead purifications were carried out, respectively, with a bead-to-sample volume ratio of 0.95x and 1x. For the 4SU/mRNAseq experiment, 200ng of purified 4SU labelled RNA per sample were used to prepare polyA+ mRNA libraries with KAPA mRNA HyperPrep kit (Roche, 08098123702) following manufacturer instructions. The libraries were then sequenced with single end 75bp reads on the Hiseq2500, with 50,000,000 reads per sample.

#### Mass spectrometry analysis

Cells were exposed to HS for 2 hours and let recover at 37°C either for 3 hours (T1) or 6 hours (T2). Cell pellets were lysed with lysis buffer (1% (w/v) Sodium Deoxycholate, 100 mM Tris, pH 8.5) and incubated for 10 min at 95°C followed by sonication using a Bioruptor pico (30 cycles, 30s on/off, ultra-low frequency). Samples were cleared by centrifugation reduced with 5 mM of TCEP for 15 min at 55°C and alkylated with 20 mM CAA for 30 minutes at RT. 100 μg of reduced and alkylated lysates were digested adding Trypsin/LysC 1:100 (enzyme/protein ratio). Peptides were cleaned up using StageTips packed with SDB-RPS resin, and resuspended in 50 μl TEAB 100 mM, pH 8,5. 50 μg of each sample was labeled with 0.5 mg TMTpro labeling reagent according to the manufacturer’s instructions. Labeled peptides were combined and cleaned up using C18-E (55 μm, 70 Å, 100 mg) cartridges (Phenomenex). Labeled desalted peptides were resuspended in Buffer A^∗^ (5% acetonitrile, 1% TFA), and 30 μg fractionated into 24 fractions, by high-pH fractionation (Kulak et al., 2017).

Each fraction was measured on an EASY-nLC 1200 ultra-high-pressure system coupled through a nano-electrospray source to a Tribrid Eclipse mass spectrometer (Thermo Fisher Scientific). Peptides were loaded in buffer A (0.1 % formic acid) onto a 25 cm Aurora HPLC column (Ionopticks) and separated with a non-linear gradient of 5 – 44 % buffer B (0.1 % formic acid, 80 % acetonitrile) at a flow rate of 400 nl/min over 91 min. The column temperature was kept at 40° C. Data acquisition switched between a full scan (120 K resolution, 50 ms max. injection time, AGC target 100%) and data-dependent MS/MS scans (50K resolution, 120 ms max. injection time, AGC target 200%) over a fixed cycle time of 3 s. The isolation window was set to 0.7 (m/z), and normalized collision energy to 35. Precursors were filtered by precursor envelope fit of 70% with fit window of 1.2 m/z, charge state of 2-6 and multiple sequencing of peptides was minimized by excluding the selected peptide candidates for 45 s.

#### Purification of bovine RNAP II from calf thymus

BOVINE RNAP II was prepared as previously described with minor modification (Zatreanu et al., 2019). Unless otherwise noted, all steps were completed at 4° C. Calf thymus was homogenized for 3 min in buffer A (50 mM Tris, pH 7.9 at 4 °C, 10 μM ZnCl_2_, 10% glycerol, 1 mM EDTA, protease inhibitors) using a 2L blender (Waring). The homogenized material was centrifuged and the supernatant filtered through two layers of mesh nylon filter. A 10% solution of polyethyleneimine, pH 7.8, was added to a final concentration of 0.05%, and the material was stirred for 30 min, then centrifuged for 30 min at 12,000g. The resulting pellets were redissolved in buffer B (50 mM Tris, pH 7.9 at 4° C, 10 μM ZnCl_2_, 10% glycerol, 150 mM (NH_4_)_2_SO_4_, 1 mM EDTA, protease inhibitors). After centrifugation, the supernatant was loaded on a 120 mL Fast Flow Q Sepharose column (Cytiva 17-0510-01), equilibrated in buffer B, by using a peristaltic pump at 5 ml/min. The column was washed with three column volumes of buffer B, followed by elution with buffer C (50 mM Tris, pH 7.9 at 4° C, 10 μM ZnCl_2_, 500 mM (NH_4_)_2_SO_4_, 1 mM EDTA, protease inhibitors). The material was further purified using a 5 mL gravity flow column of 8WG16 (αRPB1 CTD) antibody-coupled Sepharose. The input was loaded overnight using a peristaltic pump at 0.1 ml/min. After application of the input material, the antibody column was washed with ten column volumes of buffer C, sealed, and allowed to equilibrate to room temperature (20–25° C) for 15 min. RNAP II was eluted using elution buffer (40% 1,2 propanediol, 500 mM (NH_4_)_2_SO_4_, 50 mM Tris pH7.8, 10 μM ZnCl_2,_ 1 mM EDTA), collecting 4x 10 mL fractions at room temperature. The RNAPII containing fractions were dialyzed against dialysis buffer (50 mM Tris pH 7.8, 10 μM ZnCl_2_, 5 mM DTT, 10% glycerol, 150 mM (NH_4_)_2_SO_4_) and concentrated using a 100-kDa cut-off Amicon concentrator (MerckMillipore, UFC8100) to a final concentration of around 1 mg/ml.

#### In vitro transcription assay

The elongation complex was assembled as previously described with minor modification (Han et al., 2017). First, 5 pmol of pre-annealed RNA:DNA (template strand) hybrid was mixed with equimolar amount of RNAPII, followed by adding 10 pmol of 5’ biotin-labelled non-template strand DNA. The assembled elongation complex was then immobilized onto streptavidin beads (Dynabeads MyOne Streptavidin T1 (Invitrogen, 65601) and washed with transcription buffer (TB) containing 20 mM Tris-HCl pH 7.5, 100 mM NaCl, 8 mM MgCl_2_, 10 μM ZnCl_2_, 10% glycerol, 2 mM DTT, then with TB/0.1% Triton, TB/0.5 M NaCl and finally TB.

A long transcription template (around 1 kb) was PCR-amplified from RPL41 termination region by using primers containing BbsI restriction digestion sites. The PCR product was then digested with Bbs I and ligated to the pre-assembled RNAPII elongation complex by T4 DNA ligase.

After ligation, the transcript was first pulse radiolabelled with alpha-32P UTP, then chased with 100 μM NTPs in transcription buffer under 37° C vs 43° C. Reactions were stopped at indicated timepoint by mixing with 25 mM EDTA (final concentration) and separated into supernatant and bead fraction. The bead fractions were resuspended in 8 μl of loading buffer (1× TBE, 8 M urea) and boiled for 5 min at 95° C, and RNAs in the supernatant fractions were ethanol precipitated and resuspended in 8 μl of loading buffer. The samples were subjected to 6% (w/v) denaturing PAGE (8 M urea) and the gel was visualized by phosporimaging using a Typhoon scanner (GE healthcare).

RNA: UUUUUCGACCAGGA

hplc purified from IDT

Template DNA: containing 5’ phosphor modification in order to ligate to long transcription templates

GTTGTGCAGGCCGGGTGCGGCCGCCCGTGTGGAGATGGGTGAGAGATGTTGAGGATCCTGGTCGTTTCCTATAGTTTGTTTCCTGAGTAAGTCTTCATCG

page purified from IDT.

Non-template DNA:

CTAGCGATGAAGACTTACTCAGAAACACGACTTAGGTAGACGACCAGGATCCTCAACATCTCTCACCCATCTCCACACGGGCGGCCGCACCCGGCCTGCA

page purified from IDT.

### Quantification and statistical analysis

#### TT_chem_-seq alignment and quantification

Illumina adapter sequences were trimmed from reads using cutadapt v1.18 (Martin, 2011) with the following settings: -a AGATCGGAAGAGC -e 0.1 -q 10 -m 25 -O 1. Reads were aligned against the Homo sapiens GRCh38 genome build using STAR v2.5.2a (Dobin et al., 2013) with Ensembl release 86 transcript annotations. A yeast spike-in was used to normalise sequencing depth between samples. Scale factors were derived by aligning reads to the Saccharomyces cerevisiae sacCer3 genome build and generating a gene counts matrix using the standard Ensembl gene definitions. The counts matrix was then passed to DESeq2’s estimateSizeFactors function (Love et al., 2014). Note that yeast scale-factors were applied to all TT_chem_-seq datasets except for the mRNA and the CPSF73-dTAG datasets, where the default human factors were used.

#### Differential expression

Human gene abundance estimates were calculated using the 3’ 50% of the gene-level intervals using the GenomicAlignments (Lawrence et al., 2013) package’s summarizeOverlaps function (mode=”Union”, ignore.strand=FALSE). Yeast derived scale replaced the default size factors from the analysis of the human data. Differential expression analysis was conducted between treatment groups using the Bioconductor package DESeq2 (Love et al., 2014). Genes were filtered for significance based on an FDR<=0.01, a fold-change of +/- 2, and a base-mean expression of >=10.

#### BAM files merging

For the purposes of visualisation, genome alignment BAM files were merged across biological replicates, sorted and indexed using Picard v2.1.1 (http://broadinstitute.github.io/picard).

#### BigWig files

BigWig files were generated by converting BAM files to bedGraph format using BEDtools’ genomecov function (Quinlan and Hall, 2010) in a strand-specific manner. Where applicable, yeast scale factors were applied to normalize for differences in library size. bedGraph files were in turn converted to bigWig format using the bedGraphToBigWig function from the KentTools package (Kent et al., 2010). All the genome-wide visualizations are strand-specific and represent a merge of at least two biological replicates.

#### Read depth profiles

Read depth profiles were created directly from the scaled bigwig files using deepTools v.2.5.3 (Ramírez et al., 2014) for protein coding genes from standard chromosomes that were between 5-300kb in width. Additional gene sets were created by sub-setting genes based on their genomic width: >=5kb, >=30kb, >=60kb, >=90kb and >=120kb and also on differential expression status.

For the metagene profiles the computeMatrix function was used to calculate read coverage over the regions 5kb upstream of the TSS, the gene-body divided into 100 equally sized bins and the 5kb downstream of the TSS (computeMatrix scale-regions -m 15000 -a 5000 -b 5000 --binSize 100). For the CPSF73-dTAG dataset, the metagene profiles were additionally transformed to bring the curves into alignment and better highlight relative changes in expression across the genic interval. This was done by scaling each curve by the sum of sense coverage upstream of the TSS, effectively aligning the y-axes based on the promoter reads which is presumed to be background signal.

For the extended TSS profiles the computeMatrix function was used to calculate read coverage over the regions 2kb upstream to 120kb downstream of the TSS (computeMatrix reference-point --referencePoint TSS -a 120000 -b 2000 --binSize 100).

Coverage matrices were imported into R for further processing and visualisation. The mean read-depth at each bin was calculated across contributing genes having trimmed the 5% most extreme values.

#### Travel ratios

Travel ratios were calculated as in Rahl et al. (2010). Briefly, each gene was divided into i) a promoter-proximal bin −30 bp to +300 bp around its TSS and ii) a gene body bin extending to the TTS. The trave ratio is the ratio of RNAPII density in the promoter-proximal bin to that in the gene body. A count of 1 was added to the promoter and gene body counts prior to calculating density in order to mitigate for zero and infinite travel ratios. All protein coding genes from standard chromosomes that were between 5-300kb in width were considered for the analysis, but subsets were taken corresponding to genes significantly up or down regulated between heat-shock and control samples based on the DESeq2 analysis.

#### DRB/TT_chem_-seq: wave-front and elongation rate

DRB/TT_chem_-seq analysis was conducted as in Gregersen et al. (2020). Briefly, bp-resolution read-depth coverage was calculated over intervals representing non-overlapping, protein-coding genes at least 200kb in width, extended -2kb:+200kb around the TSS region. Scale factors derived from the yeast spike-in were used to normalise counts for library size before being converted to read-counts-per-million (RPM). Meta-profiles were created by taking a trimmed mean across genes at each bp location. A smoothing spline was fitted to each meta-profile using R’s smooth.spline function (spar=0.3). Wave peaks were subsequently calculated as the maximum point on the spline for each sample, ensuring that wave peaks advanced with time. Elongation rates in kb/min were estimated by fitting a linear model to the wave peak positions of all samples (i.e. 0, 10, 20, 30, and 40 minutes post release) as a function of time.

#### FP/TT_chem_-seq: wave-front and elongation rates

Extended TSS (-2k:+200kb) sense read depth profiles were smoothed using a spline. The slope of the transcriptional wave indicative of polymerase speed was defined as the point at which the curve was approximately linear. This was estimated by calculating the gradient of the first differential at the point the second differential crossed zero.

Exemplar single gene level wave positions were calculated from scaled read-depth coverages converted to densities over the region TSS:+200kb. The wave was defined at the position at half the height of the maximum observed density within the first 150kb. Elongation rates in kb/min were estimated by fitting a linear model to the wave peak positions of all samples (i.e. 0, 15, 20, 25, 40 and 40 minutes post release) as a function of time.

#### FP/TT_chem_-seq: read-through-ratio

The read-depth-ratio was defined as the ratio of read density of terminal exons divided by the read density in the downstream 20kb region. Only protein coding genes from standard chromosomes were considered. A single representative transcript was selected per gene, prioritising better transcript support level then larger transcript width.

#### TTchem-seq: defective transcript elongation after heat shock

Non-overlapping, protein-coding genes were divided into two intervals: a 5’ region representing the first 5%’ of a gene’s width downstream of the TSS and a 3’ region representing the final 25% of the gene up to the TES. Sense reads were counted over these intervals and converted to densities by dividing by interval width. Genes containing an interval with zero counts in either the HS or ctrl condition were discarded, leaving n=4876 genes. A ratio of each gene’s 3’ and 5’ interval densities was calculated for each treatment condition. Finally, a ratio-of-ratios between the HS and ctrl samples was calculated: HS(3’/5’)/ctrl(3’/5’). The data were stratified based on gene width into 6 groups (<15kb, 15<30kb, 30<60kb, 60<90kb, 90<120kb, >=120kb) and the number of genes showing at least a 1.5-fold reduction in each width group was recorded.

#### mRNA-seq first exon enrichment analysis

This analysis attempted to measure the enrichment of expression of short isoforms within HS or Ctrl treatment groups by comparing the number of reads mapping to first exons relative to all remaining exons. A single transcript was selected per gene (Gencode Basic v33) as being representative of each locus, giving priority to transcripts with the strongest support level and then by longest genomic interval. Genes comprising just a single exon were discarded. Exon level read counts were generated from BAM files using the GenomicAlignments package’s summarizeOverlaps function in a strand-specific manner (ignore.strand=FALSE, inter.feature=TRUE). The matrix was used to generate sample scale factors using DESeq’s estimateSizeFactorsForMatrix function, with default settings. The exon level counts matrix was then recoded such that there were 2 paired columns per samples representing i) first exon counts and ii) remaining exon counts. This new matrix was further analysed using DESeq2. Size factors generated from the above all exon analysis were applied to this new matrix such that the same size factor for a given sample was applied to both the “first exon” and “remaining exon” columns. DESeq2’s Wald test was then run with the following design formula: ∼ HS_status + HS_status:pair + HS_status:sample. The resulting logFC from the test equates to the ratio of first/remaining exon counts for each treatment, i.e. (HS first/ HS remaining) / (CTRL first/CTRL remaining). Thus, a positive logFC indicates a preference for shorter isoforms in the HS relative to the CTRL and a negative logFC indicates a preference for shorter isoforms in the CTRL relative to the HS. Figure 3B depicts these logFC values for all expressed genes, i.e. a count of at least 1 in i) exon1 and ii) remaining exons across all HS and ctrl samples (n=8744).

#### Genomic features analysis

The shrunken-logFC estimates from the DESeq2 analysis of the TT_chem_-seq MRC5-VA differential expression analysis were used to assess treatment-specific differences associated with various genomic features. In each case, the logFC estimates were stratified on gene differential expression status in the HS samples relative to the control and presented as boxplots. Gene width was defined as the genomic interval from TSS to TES. U1 motifs and polyadenylation site (PAS) analysis was conducted as in Krajewska et al. (2019). Briefly, U1 motifs (“GGTGAG”, “GGTAAG” or “GTGAGT”) and PAS motifs (“AATAAA”) were counted within the genic intervals. The “U1/PAS” ratio was defined as the ratio of these counts, i.e. (GGTGAG + GGTAAG + GTGAGT)/(AATAA). Significance differences in the distribution of logFCs between HS down-regulated genes and all other genes was assessed using a Wilcoxon rank sum test.

Gene-width was plotted against the shrunken-logFC estimates from the DESeq2 analysis of the HS vs ctrl samples for all protein-coding genes from standard chromosomes. Genes significantly up and down regulated in the HS samples were highlighted. A loess line was fitted to the data to highlight the trend for down-regulated genes to be generally wider.

Abundance estimates for the HS and ctrl samples were plotted against the mean-centred log_2_ gene-widths. Abundance was rlog transformed to minimises differences between samples for genes with small counts and to normalise with respect to library size. Linear regression was applied to calculate gene-width coefficients defined by the slope of each line. A t-test was then used to assess the significance of differences in the coefficients due to the HS treatment.

#### mRNA-seq first exon enrichment analysis (U2OS cells)

Published RNA-seq data from untreated U2OS cells and those exposed to heat stress (43^o^C) for 4 hours were downloaded from ArrayExpress under accession E-MTAB-2349. Reads were aligned to the hg38 genome as per the previously mentioned TTchem-seq methodology. First-exon enrichment analysis was conducted as for the MRC5-VA cells.

#### HEK293 Pro-seq data analysis

Published HEK293 Pro-seq data were downloaded from the NCBI Gene Expression Omnibus (GSE112379) (Aprile-Garcia et al., 2019). Reads were trimmed to remove adapter sequence using trimGalore v0.4.4 (trim_galore --quality 20 -e 0.05 -a TGGAATTCTCGGGTGCCAAGG --length 10) before alignment to GRCh38 using HiSat2 v2.0.4 allowing for up to 5 primary alignments (k=5). Aligned reads were filtered to remove low quality alignments using SAMtools (Li et al., 2009) [-q 20] and used to create strand-specific BigWig files of the 5’ positions of each read. Metagene profiles were created covering the region 500bp downstream of the TSS to the TES to focus attention on the nascent RNA differences over gene bodies. No correction for differing library size between samples was performed.

#### Correlation of HS expression and U1 induced PCPA sites

Gene Set Enrichment Analysis (GSEA) was used to assess whether there was a significant, concordant difference in HS responsive gene expression in genes previously shown to exhibit premature cleavage and polyadenylation (PCPA) sites after 8h of U1 inhibition (Oh et al., 2017). Genes from the TTchem-seq differential expression analysis were ranked ordered based on their DESeq2 Wald test statistic generated from the comparison of heat-shock to untreated samples. The expression related enrichment of U1 responsive genes within that list was assessed using the Bioconductor package “fgsea” (Korotkevich et al., 2019).

#### Computer modeling of transcription

Adapting the approach previously described (Tufegdzic Vidakovic et al., 2020), we simulated polymerase dynamics in response to a discrete set of events (including initiation, termination, processivity errors - for a full list see Tufegdzic Vidakovic et al. [2020]) happening in continuous time in model cells expressing a mix of short (5kb), medium (63kb) and long (100kb) genes.

To model the behaviour of transcription after HS, where transcription ends much earlier in genes on a genome-wide scale, we replaced the stochastic damage-and-repair mechanism from Tufegdzic Vidakovic et al. (2020) with a fixed site at 10 kb into the model genes upon reaching which, polymerases will terminate and be recycled for new transcription.

In the model, a dynamic equilibrium is first achieved under the normal conditions of cells having the mix of active short (5 kb), medium (63 kb) and long (100 kb) genes as in Tufegdzic Vidakovic et al. (2020). New HS-induced transcription termination sites (TSSs) are then for simplicity introduced at 10 kb for all genes, at which point longer genes start to lose polymerases at both the new 10 kb TSS and at the previously normal TTS (at 63 or 100 kb), until the region between them is devoid of active polymerases. The model assumes that the RNAPII pool is limiting and that transcription initiation is attempted following a Poisson process whose rate is proportional to the number of free polymerases, with the constant of proportionality being such that expected times between transcription events on a gene is 2.5s in normal conditions. We then plot how the average density (100 simulation runs, arbitrary scale consistent across runs and gene-lengths) of active polymerases changes across time from the instantaneous introduction of the 10 kb TSS. The increase in transcription rate, enabled by the larger pool of free RNAPII, is most easily deduced from the initiation point on the Y-axis. The Julia code to recreate this is at Github (https://github.com/FrancisCrickInstitute/babs_uv_polymerase/releases/tag/v0.1.0).

#### Data analysis, mass spectrometry

##### Short peptide database

A library of short peptide sequences for mass-spectrometry screening was created based on Gencode (v33) non-overlapping, protein-coding transcripts from standard chromosomes. A simple, relatively straightforward approach was taken to avoid the considerable complexity of assembling a brand-new transcriptome, gene-by-gene, based on the thousands of individual new mRNA transcripts that had been revealed by our mRNA analysis. In this virtual library, translation starts at the canonical ATG of every gene considered in the analysis and is then allowed to proceed through the first exon, into the adjacent intron, and up to the first stop codon (examples shown in Figure S9). It thus assesses only incompletely but proteome-wide, the likelihood that the new mRNAs produced after HS are translated efficiently.

Raw mass spectrometry data were analyzed with MaxQuant (v1.6.15.0). Peak lists were searched against the human Uniprot FASTA database combined with 262 common contaminants by the integrated Andromeda search engine. The false discovery rate was set to 1 % for both peptides (minimum length of 7 amino acids) and proteins.

All statistical analysis of TMT derived protein expression data was performed using the automated analysis pipeline of the Clinical Knowledge Graph (Santos et al., 2021). Protein entries referring to potential contaminants, proteins identified by matches to the decoy reverse database, and proteins identified only by modified sites, were removed, as well as proteins not found in all 9 channels (samples). Reporter ion intensities were log_2_ transformed and normalized to adjust the TMT experiment to an equal signal per channel (equal mean in all channels).

Principal component analysis (PCA) was performed and visualized with 2 principal components, showing clear separation between control samples and the other groups. Differentially expressed proteins were identified with two different methods: One-way Anova followed by pairwise comparison posthoc tests (unpaired t-tests) with Benjamini-Hochberg correction for multiple hypothesis (alpha=0.05, Fold Change (FC)=1.5); and SAMR Multiclass test followed by pairwise comparison posthoc tests (two class unpaired tests) with permutation-based FDR correction (FDR = 0.05, s0=1, permutations=250, FC=1) (Tibshirani et al., 2018). Significantly regulated proteins are colored in red and blue in the volcano plots for up and downregulated hits, respectively. Hierarchical clustering was performed using all significant proteins (up- and down-regulated). The selected proteins were then mean normalized.

Functional enrichment analysis was performed in the Clinical Knowledge Graph [2], applying Fisher’s exact test and Benjamini-Hochberg FDR correction, and using Gene Ontology Biological Process (GOBP), Gene Ontology Molecular Function (GOMF), Gene Ontology Cellular Component (GOCC) and Reactome annotations. All proteins quantified in the experiment were used as background, and statistically significant proteins (posthoc tests) were considered foreground. Enrichment analysis is performed for up- and down-regulated proteins separately.

## Data Availability

Raw FASTQ files and unscaled bigwig files were deposited to the NCBI’s Gene Expression Omnibus under accession number GEO: GSE165368. The original images of the study are at Mendeley https://doi.org/10.17632/x7hcmjjpj7.1. Sequencing data have been deposited in NCBI's Gene Expression Omnibus and are accessible through GEO Series accession number GEO: GSE165368 (https://www.ncbi.nlm.nih.gov/geo/query/acc.cgi?acc=GSE165368). The code for the mathematical modelling of transcription is available at https://doi.org/10.5281/zenodo.5814979 and also at Github https://github.com/FrancisCrickInstitute/babs_uv_polymerase/releases/tag/v0.1.0.

## Supplemental information

Document S1. Figures S1–S12

Table S1. The transcriptional response to heat shock. Upregulated and down-regulated genes, related to Figure 1

Table S2. Virtual library of open reading frames designed to detect potential protein products synthesized from the new, unannotated mRNAs Translation always starts at the canonical ATG and is then allowed to proceed only through the first exon, into the adjacent intron, and up to the first stop codon (examples are shown in Figure S9). This simplified approach provides a proteome-wide overview of mRNA translation changes occurring upon HS, but also means that, for example, the translation of transcripts terminating upstream of the canonical ATG (such as the *KLHL29* gene in Figure 6) will not be included in this analysis. See also STAR Methods. Related to Figure 7

Table S3. Polypeptides detected by proteome wide mass spectrometry analysis before and after HS, related to Figure 7

Table S4. DNA primers used in this study, related to Figures 1, 2, 3, 4, 5, 6, and 7

Document S2. Article plus supplemental information
