# Supplementary material for: Heat shock induces premature transcript termination and reconfigures the human transcriptome
Source: Mol Cell. 2022 Apr 21;82(8):1573–1588.e10. doi: 10.1016/j.molcel.2022.01.007 (PMC9098121; doi:10.1016/j.molcel.2022.01.007)
Supplement: Document S1. Figures S1–S12 [file mmc1.pdf]

Molecular Cell, Volume *82*

Supplemental information

Heat shock induces premature transcript termination and reconfigures the human transcriptome

Simona Cugusi, Richard Mitter, Gavin P. Kelly, Jane Walker, Zhong Han, Paola Pisano, Michael Wierer, Aengus Stewart, and Jesper Q. Svejstrup

SUPPLEMENTARY FIGURES WITH LEGENDS

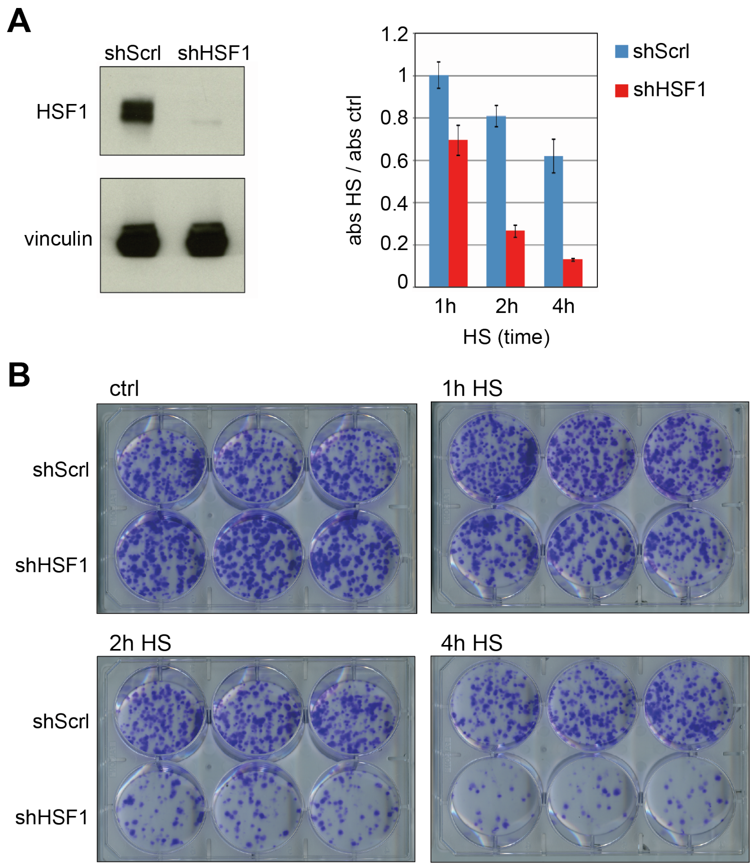

Figure S1. Cellular response to heat shock, related to Figure 1.
A. Left, western blot analysis showing HSF1 knockdown in the shHSF1 cell line. Vinculin is used as a loading control. **Right,** Crystal violet quantification from HS treated cells normalised to untreated ctrl. Average of three biological replicates, error bars indicate ±SD. **B.** Representative example of colony formation for HS survival assay (in triplicate).

A

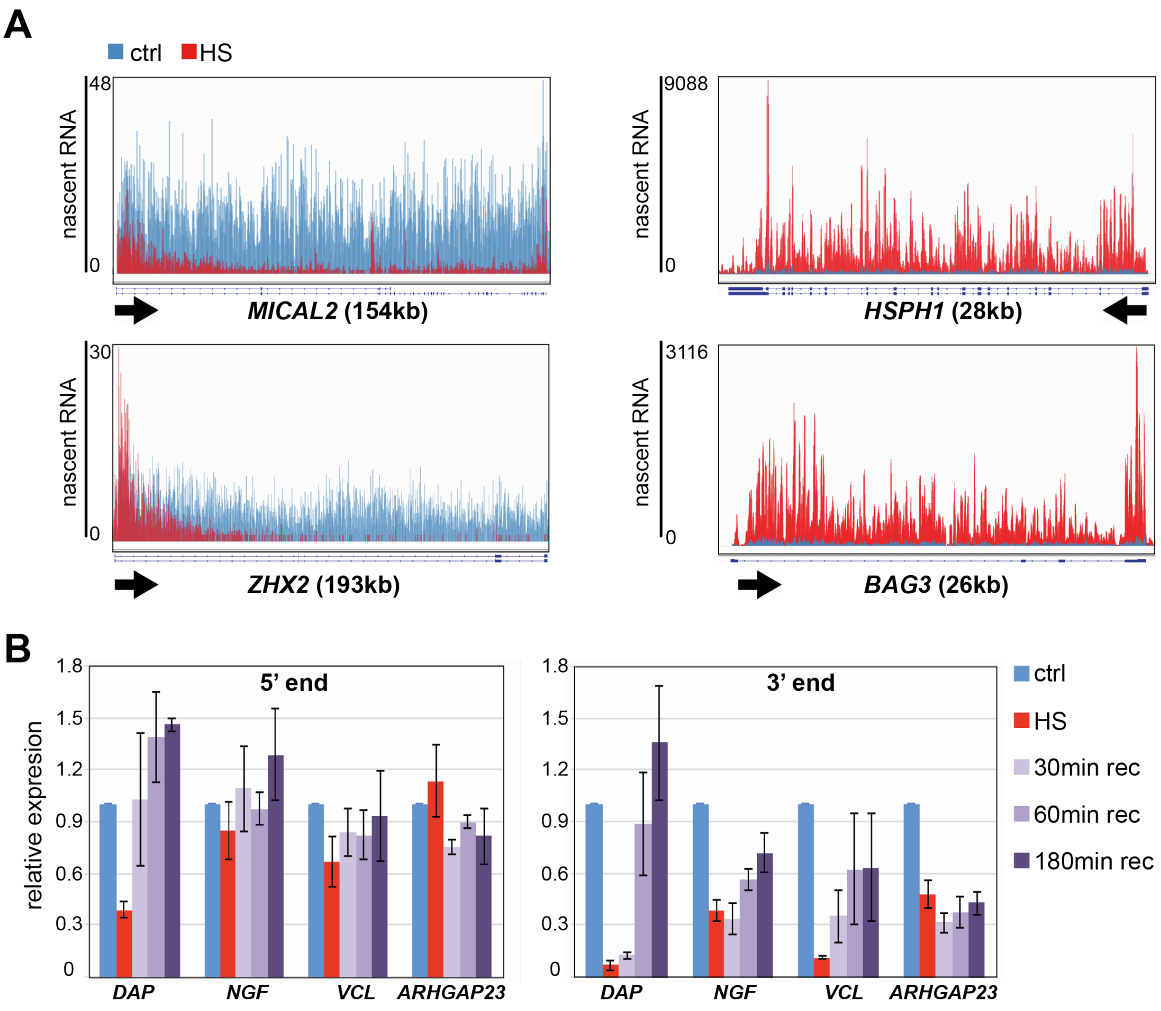

C

	width	decreased	total	perc.decreased
1	<15kb	244	771	31.65
2	15<30kb	219	701	31.24
3	30<60kb	399	972	41.05
4	60<90kb	264	546	48.35
5	90<120kb	210	385	54.55
6	>120kb	838	1501	55.83

Figure S2. Defective transcript elongation after heat shock, related to Figure 1.
A. IGV genome browser view of TT_{chem}-seq for genes down-regulated (*MICAL2* and *ZHX2*) or up-regulated (*HSPH1* and *BAG3*) after HS. **B.** qPCR quantification of nascent RNA near the 5'-end or the 3'-end of downregulated genes at various times after HS, relative to *GAPDH*, normalized to the control. Average of three biological replicates; error bars indicate ±SD. **C.** Percentage of genes downregulated >1.5 fold (activity in the 3' end of gene (last 25%) compared to first 5% of gene between conditions, *i.e.* HS(3'/5')/ctrl(3'/5')), by gene length.

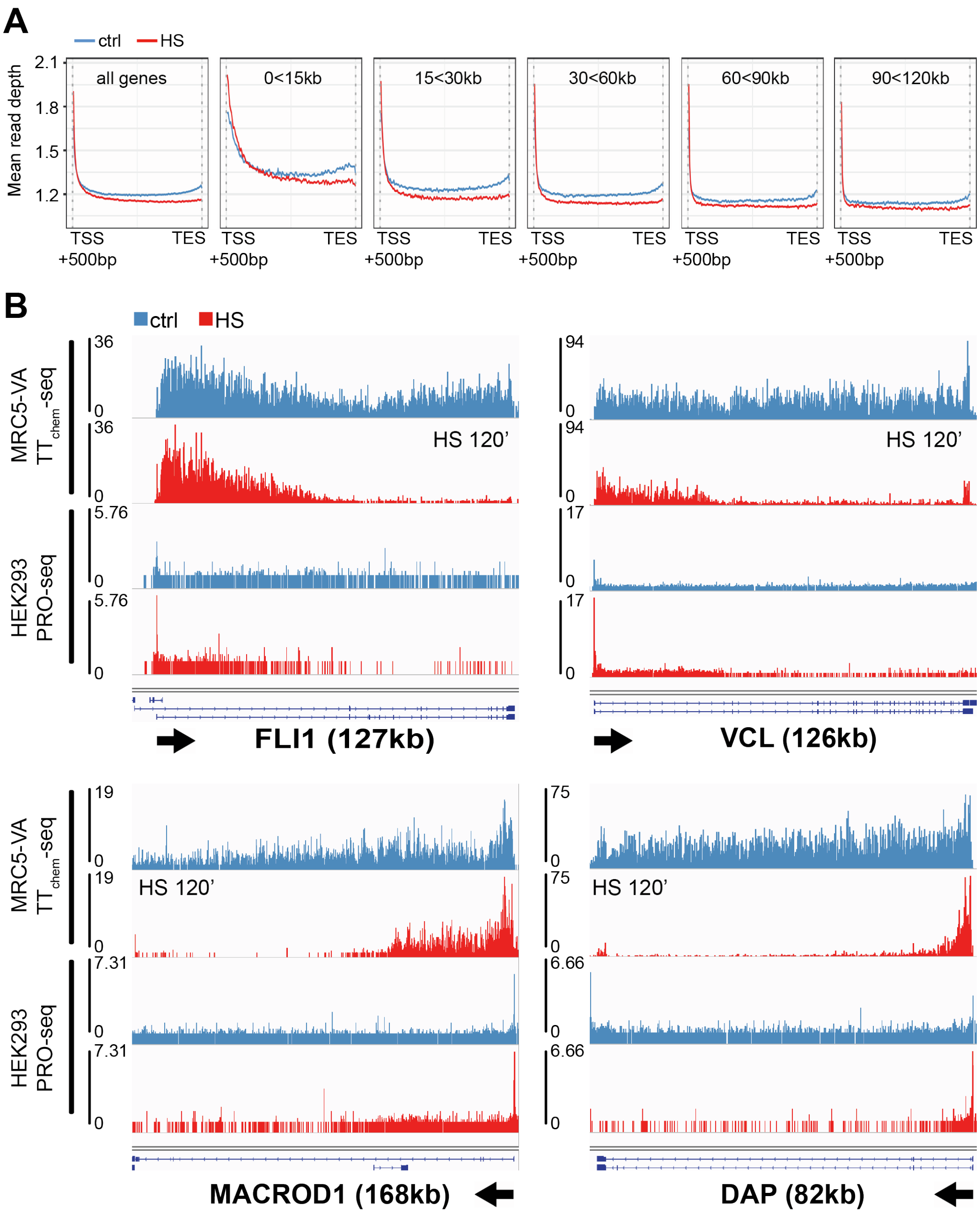

Figure S3. Defective transcript elongation after heat shock in HEK293 cells, related to Figure 1. A. Coverage plots of PRO-seq data from Aprile-Garcia et al., 2019 stratified by gene length, obtained by aligning genes at position +500 bp relative to the TSS, *i.e.*, at a point downstream from the promoter-proximal pause site. **B**. Examples of IGV genome browser views of same PRO-seq data for the *FLI1, VCL, MACROD1, and DAP* genes. Arrows indicate the direction of transcription.

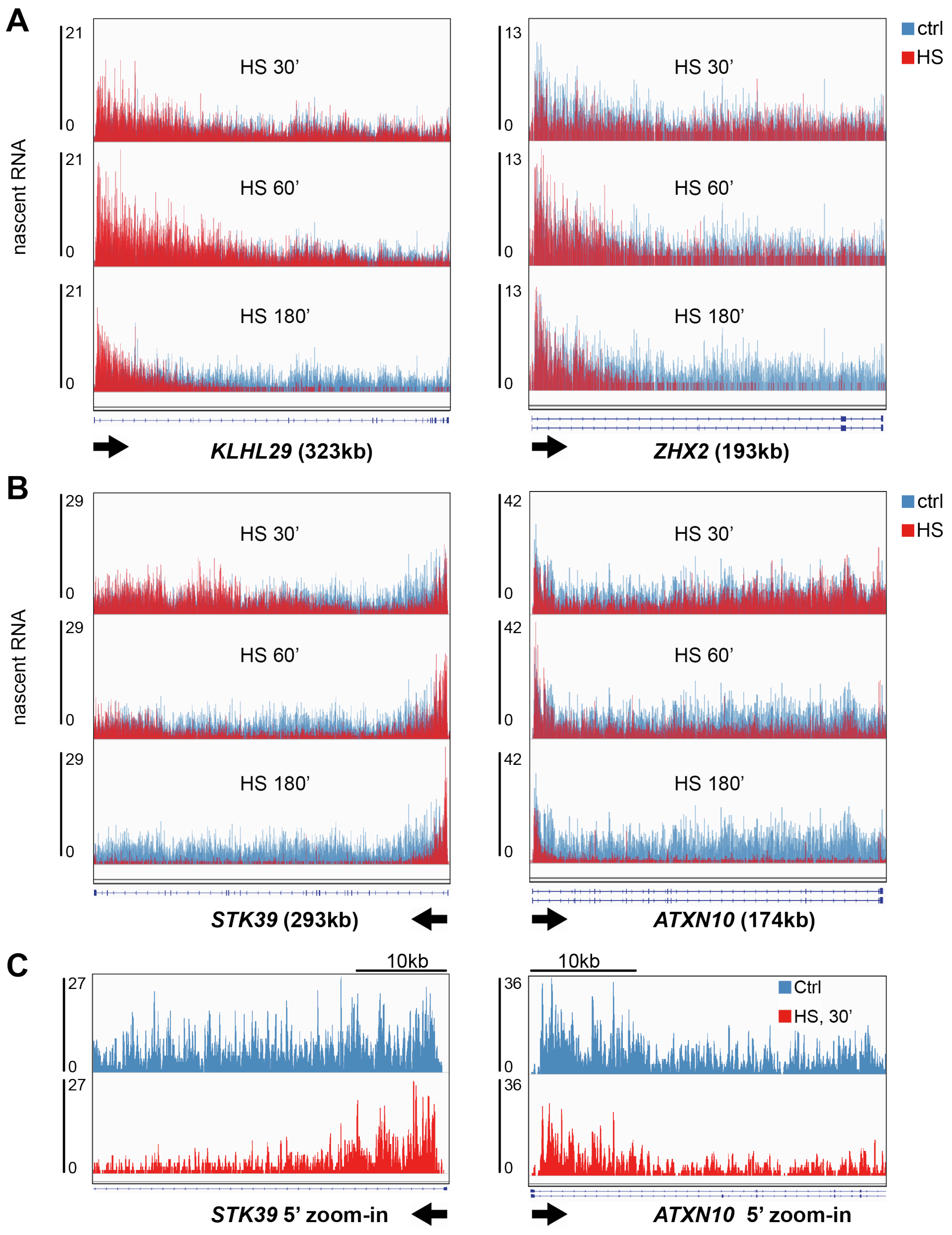

Figure S4. Transcription during a HS time course, related to Figure 2.
A. IGV genome browser view of TT_{chem}-seq for *KLHL29* and *ZHX2* in cells exposed to a HS time course. **B**. As in A, but for for *STK39* and *ATXN10*. **C.** Zoom-in detail of B, 5' gene ends.

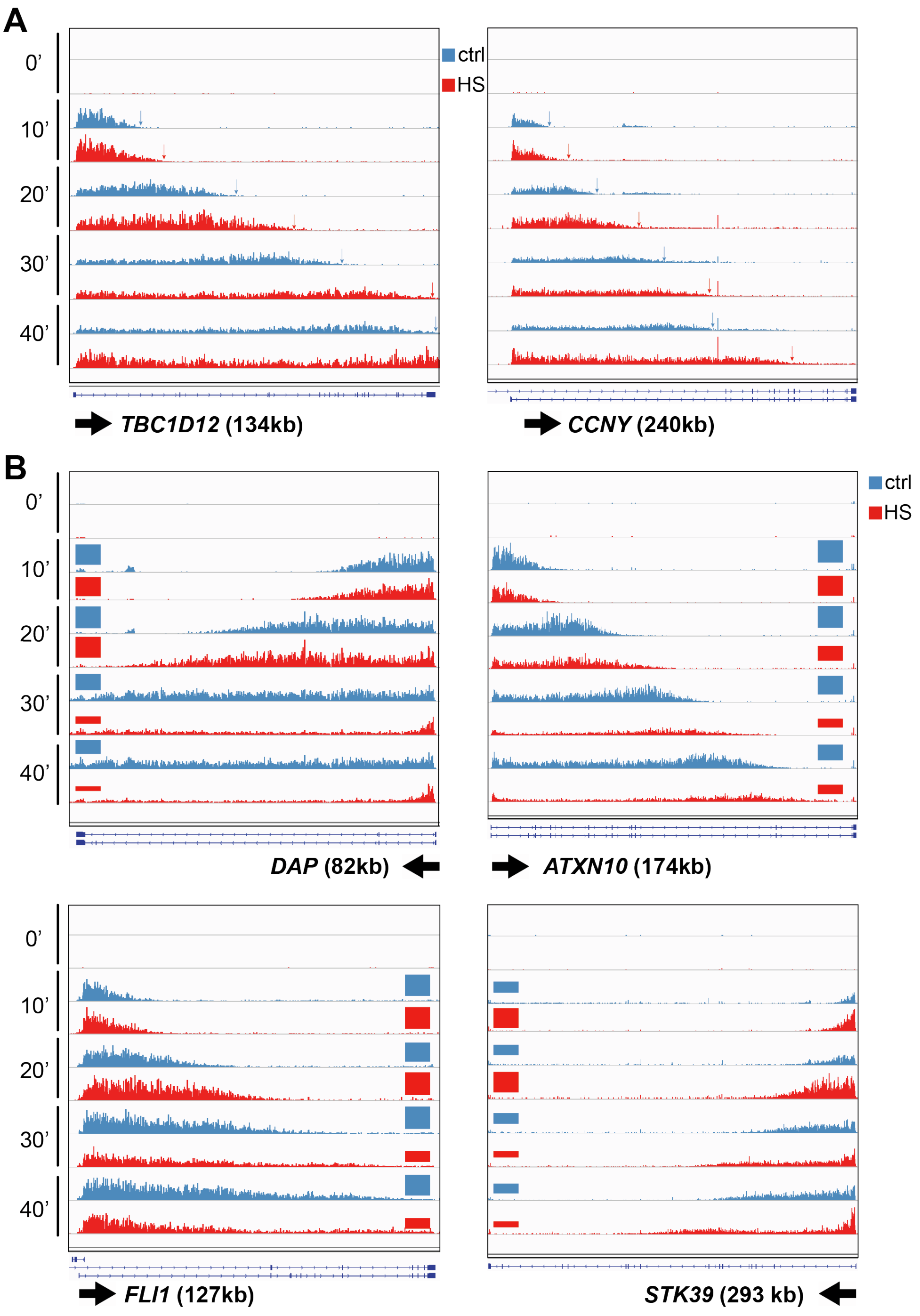

Figure S5. Transcript elongation measurement after heat shock, related to Figure 3.
A. IGV genome browser view of DRB/TT$_{chem}$-seq for the *TBC1D12* and *CCNY* genes. **B.** As in A, but for *DAP, ATXN10, FLI1* and *STK39*. Note the gradual decrease in the level of transcription activity over time in the HS-treated samples (summarized by the colored squares, positioned at the ends of genes for simplicity).

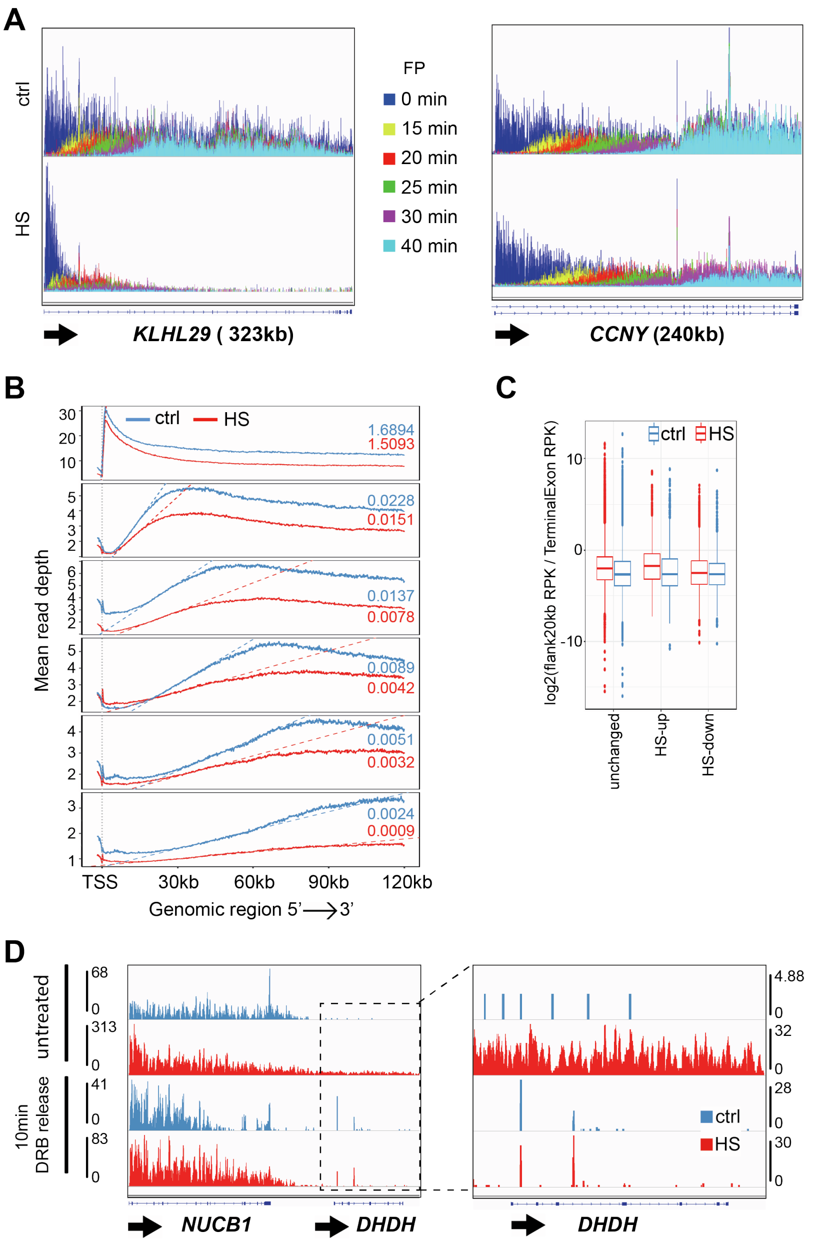

Figure S6. Transcript elongation measurement by Flavopiridol/TT$_{chem}$-seq, related to Figure 3. A. IGV genome browser view of FP/TT$_{chem}$-seq for the *KLHL29* and *CCNY* genes. **B.** Coverage plots of strand-specific FP/TT$_{chem}$-seq. The dashed lines and the numeric labels indicate the slope of the rear end of the receding transcription wave for each condition. **C.** Boxplot of readthrough ratios for HS up-regulated genes (n= 1099), HS down-regulated genes (n= 1831) and unchanged genes (16957). **D.** IGV genome browser view of TT$_{chem}$-seq ('untreated') and DRB/TT$_{chem}$-seq ('10 min DRB release'), showing readthrough into the *DHDH* gene from the upstream *NUCB1* gene. Note that no HS-independent DHDH transcription is detectable after DRB release.

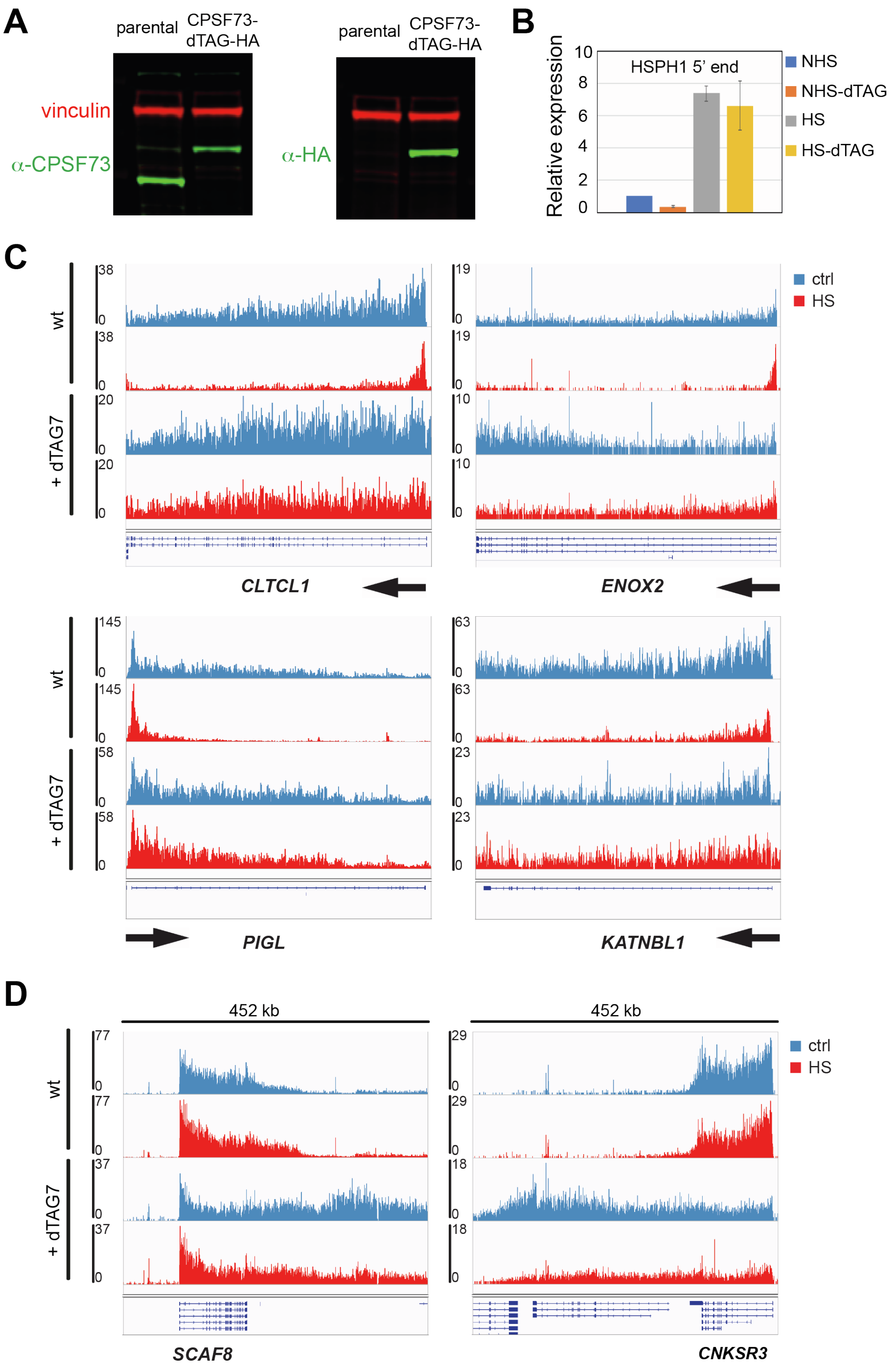

Figure S7. CPSF73 (dTAG) cell line and, related to Figure 4.
A. Western blot analysis of CPSF73 (dTAG)-HA cells. Vinculin is used as a loading control. **B.** qPCR quantification of nascent RNA near the 5'-end of the HS-*activated* HSPH1 gene, relative to *GAPDH*, normalized to the control. Average of three biological replicates, error bars indicate ±SD. **C.** IGV genome browser views of TT_{chem}-seq for 4 additional example genes in cells exposed to HS (or not), without (WT) or with CPSF73 depletion (+dTAG7). **D.** As in C, but examples of the readthrough observed downstream of canonical TESs at gene 'ends.

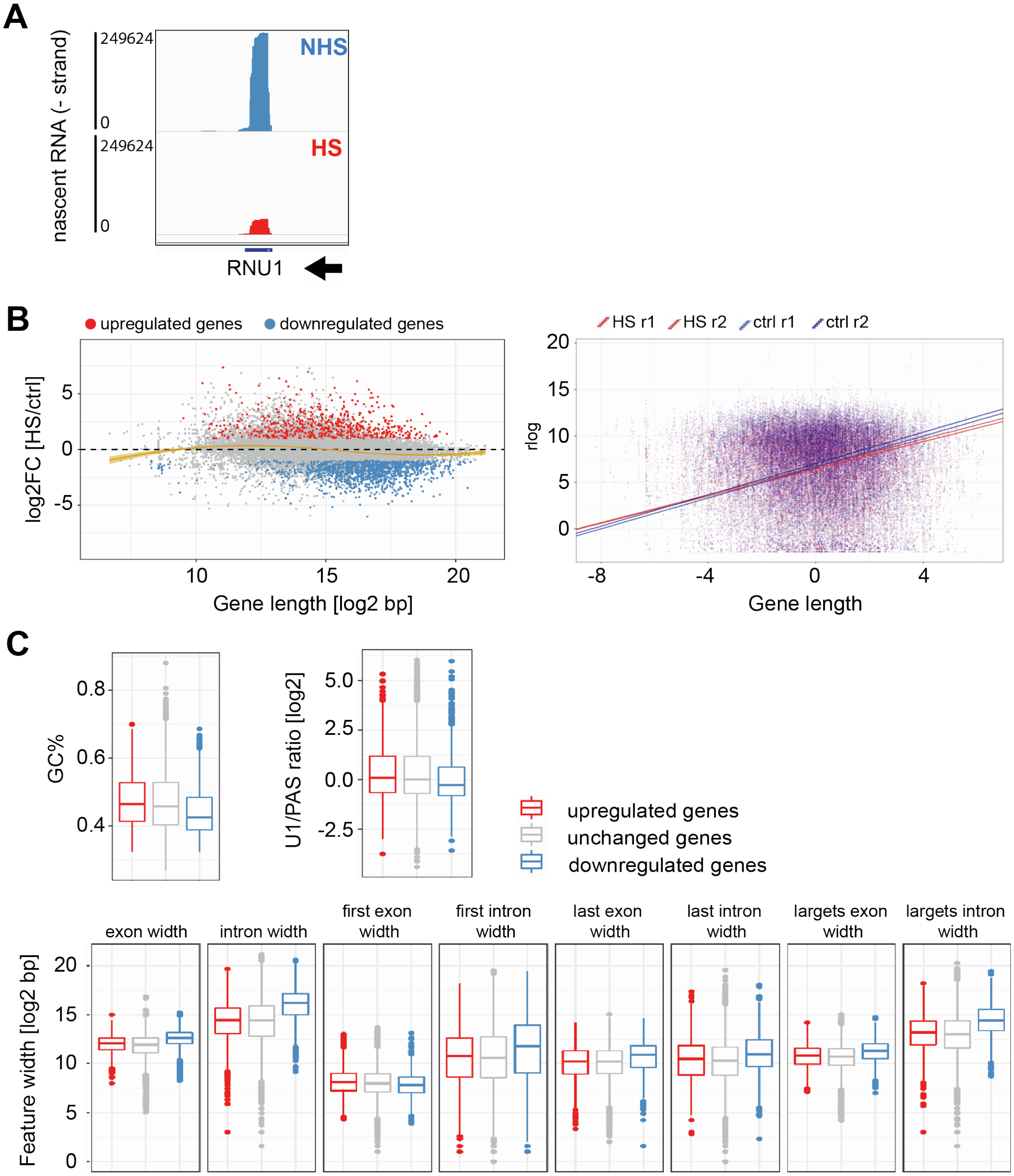

Figure S8. Genomic features of HS downregulated genes, related to Figure 5. A. IGV genome browser view of TT_{chem}-seq for the *RNU1* gene. NHS, no heat shock. **B. Left,** scatterplot showing the gene width distribution of significant up- and down-regulated genes. Unchanged genes in grey and the Loess line in yellow. **Right,** plot showing the HS dependent relationship between gene-width and transcript abundance. Gene widths were log2 transformed before being mean centred, and are shown plotted against normalised abundance (rlog) for each sample. A linear regression line was calculated through the points for each sample individually. The slope of each line yielded a gene-width coefficient that was compared between HS and CTRL conditions using a t-test. **C.** Box plots of genomic features.

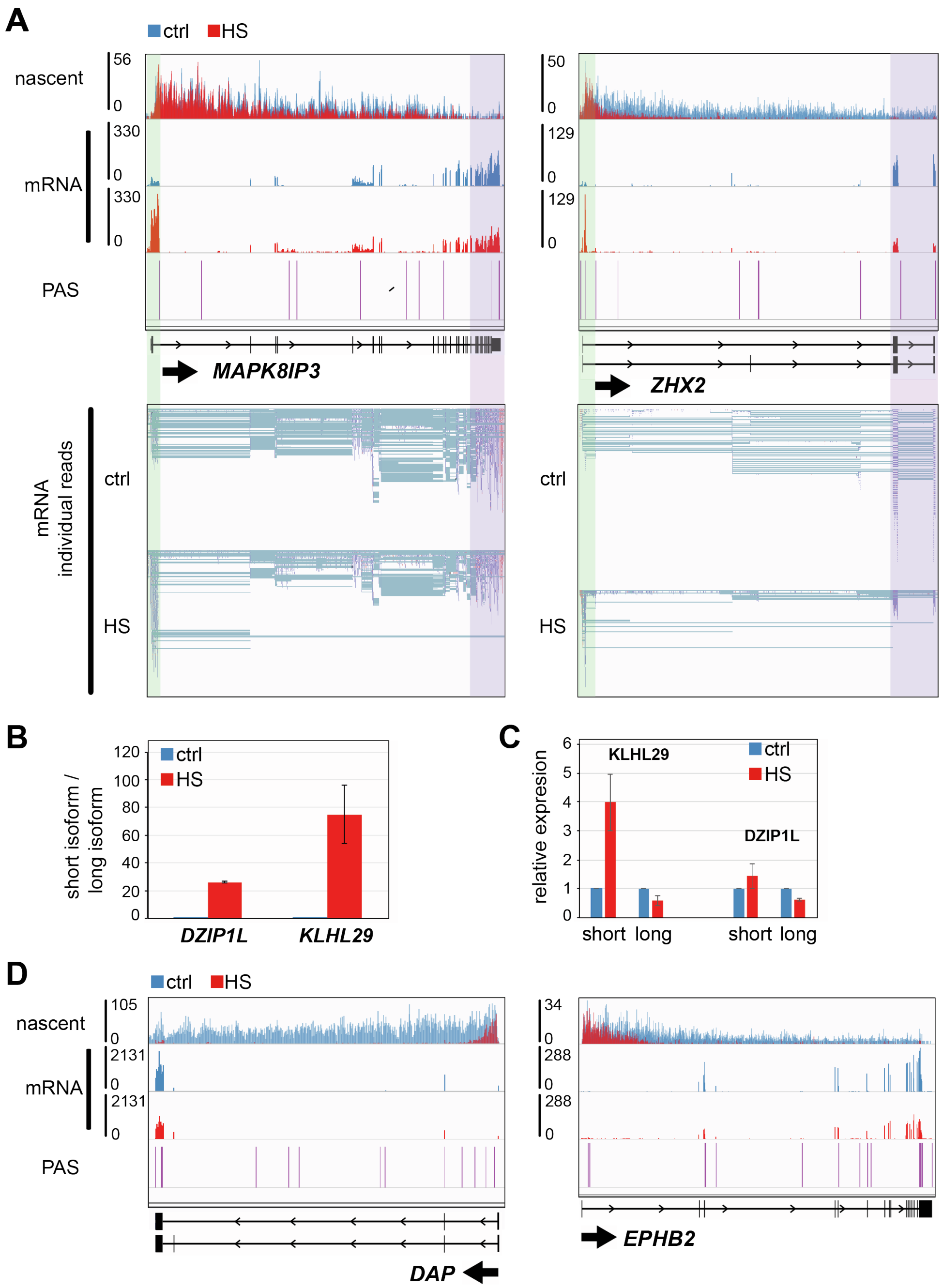

Figure S9. mRNA production after HS, related to Figure 6.
A. IGV genome browser view of TT_{chem}-seq, Neo-mRNAseq, and PASs (Wang et al, 2018) for the *MAPK8IP3* and *ZHX2* genes in MRC5-VA cells. In the mRNA individual reads (bottom panels), blue indicates the (+) strand and red the (-) strand. **B.** qPCR quantification of newly synthesized short isoform (Neo-) mRNA from *DIZIP1L* and *KLHL29*; relative to the long isoform, normalized to control. Average of three biological replicates, error bars indicate ±SD. **C.** qPCR quantification of short and long isoforms of *DIZIP1L* and *KLHL29* (total) mRNA, relative to GAPDH, normalized to control. Average of three biological replicates, error bars indicate ±SD. **D.** IGV genome browser views as in A.

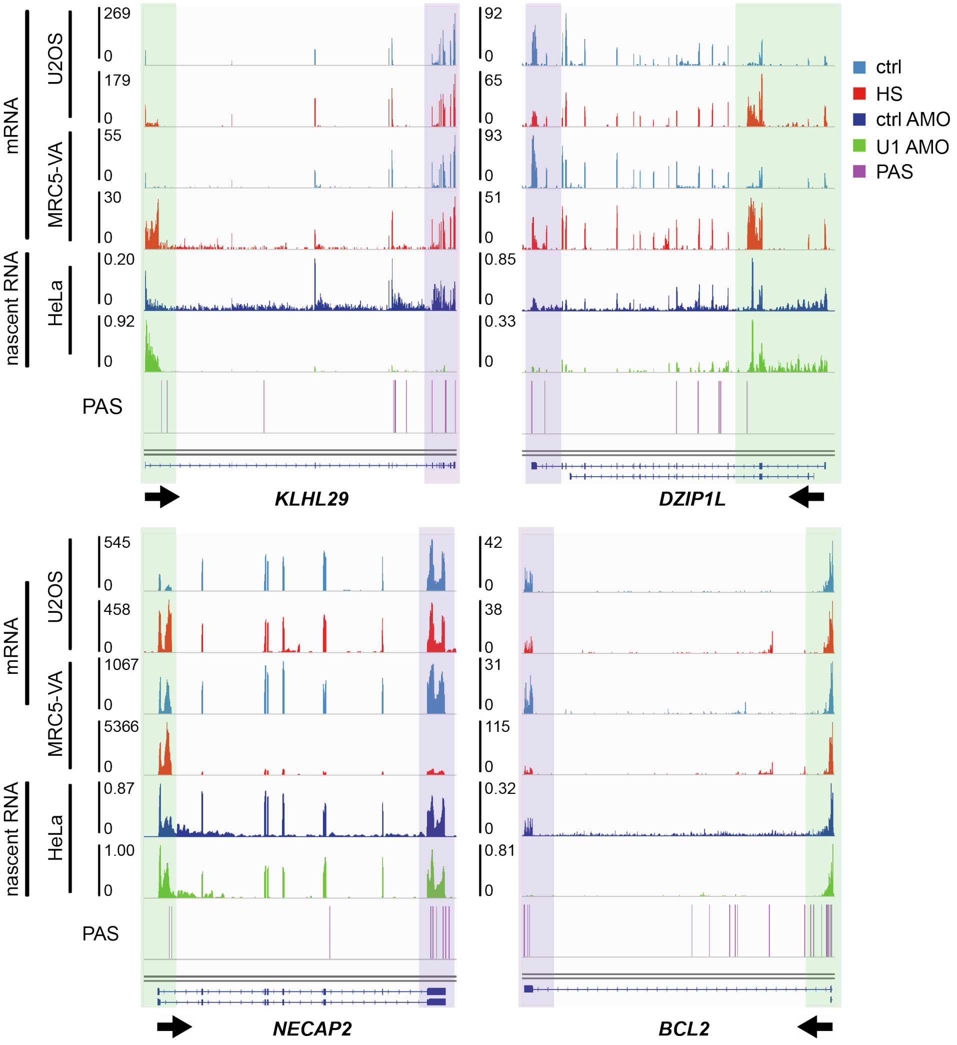

Figure S10. Transcription and mRNA production after heat shock closely resembles inhibition of U1 telescripting, related to Figure 7.
IGV genome browser views for the *KLHL29, DZIP1L, NECAP2* and *BCL2* genes, showing RNA-seq from U2OS cells (Seifert et al., 2015), Neo-mRNA-seq in MRC5-VA cells and nascent RNA from HeLa cells treated either with ctrl- or U1-AMO (So et al., 2019). PASs from the PolyA_DB database (Wang et al., 2018).

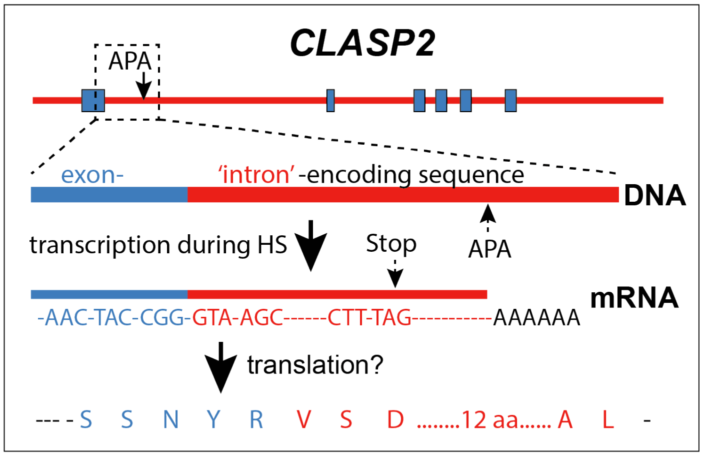

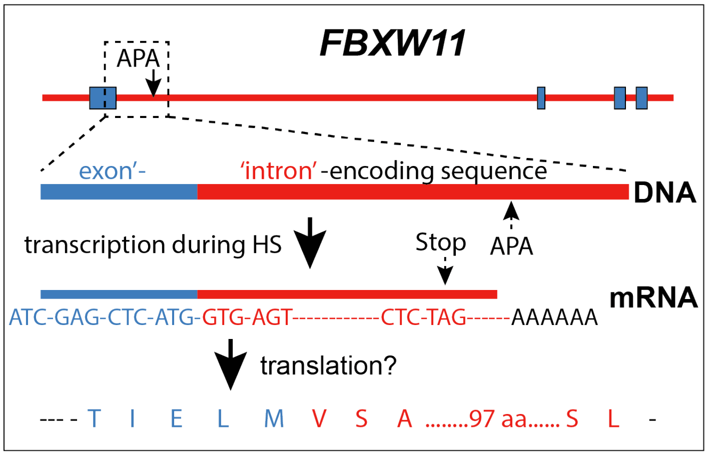

Figure S11. Novel proteins library generation, related to Figure 7.
Schematic representation of potential novel protein products for CLASP2 and FBXW11 genes.

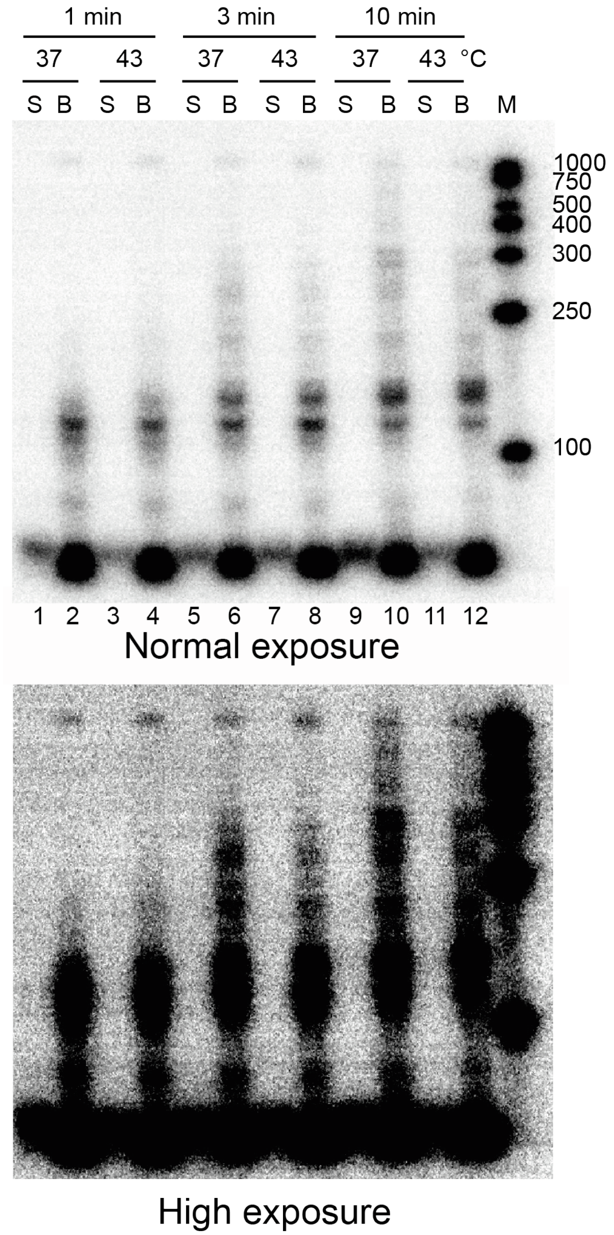

Figure S12. The transcription elongation rate is not noticeably increased at 43°C *in vitro*, related to Figure 3. Using immobilized templates, transcripts were first radioactively labeled by ATP, UTP, CTP (AUC) (with α-P^{32} UTP), then washed twice, preincubated at 37°C or 43°C for 2 min, before chasing with 100 μM (non-radioactive) AUGC for the times indicated. S, supernatant. B, immobilized transcription template. Please note that rather than increasing activity and rate, incubation at 43°C appears to be slightly detrimental under these conditions, where only purified RNAPII is present in the reaction. A similar lack of elongation rate increases at elevated temperature was observed in experiments with pure yeast RNAPII (performed at 30° (optimal growth temperature for *S. cerevisiae*), 35° and 40°C) (data not shown).
